# Deubiquitinase MYSM1 drives myocardial ischemia/reperfusion injury by stabilizing STAT1 in cardiomyocytes

**DOI:** 10.7150/thno.100097

**Published:** 2025-01-02

**Authors:** Xiaowen Shi, Jianjiang Xu, Lei Liu, Shenggang Zhao, Yuanyuan Qian, Zimin Fang, Liming Lin, Xia Zhao, Shangcai Xie, Fengjie Shi, Jibo Han

**Affiliations:** 1 Department of Cardiology, The Second Affiliated Hospital of Jiaxing University, Jiaxing, Zhejiang, China.; 2 Key Laboratory of Blood-stasis-toxin Syndrome of Zhejiang Province, Zhejiang Chinese Medical University, Hangzhou, Zhejiang, China.; 3 Department of Ultrasound, Puer People's Hospital, Puer, Yunnan, China.; 4 Department of Cardiology, the First Affiliated Hospital, Wenzhou Medical University, Wenzhou, Zhejiang, China.

**Keywords:** Myocardial ischemia/reperfusion injury, Deubiquitinating enzymes, MYSM1, STAT1, Necroptosis

## Abstract

**Rationale:** Myocardial ischemia/reperfusion (I/R) injury leads to irreversible cardiomyocyte death and aggravates myocardial infarction. Deubiquitinating enzymes (DUBs) are essential for maintaining substrate protein stability and functionality, playing significant roles in cardiac pathophysiology. In this study, we aimed to clarify the regulatory role of a DUB, Myb-like, SWIRM, and MPN domains 1 protein (MYSM1), in myocardial I/R injury and explore the molecular mechanism behind.

**Methods and Results:** Firstly, it was found that the expression of MYSM1 positively correlates with myocardial I/R injury. Genetic knockdown of MYSM1 significantly conferred protection against I/R injury in hearts. Correspondingly, AAV9-mediated cardiomyocyte-specific knockdown of MYSM1 had a therapeutic effect on myocardial I/R injury. Through a comprehensive proteome-wide quantitative analysis, we identified signal transducer and activator of transcription 1 (STAT1) as the direct substrate of MYSM1. Mechanistically, MYSM1 mediated the K63-linked deubiquitination and stabilization of STAT1 at position K379 via its MPN metalloprotease domain. Additionally, MYSM1 initiates the expression of necroptosis-related genes by promoting the transcription factor function of STAT1.

**Conclusion:** This study illustrated a MYSM1-STAT1 axis in regulating myocardial I/R injury and identified MYSM1 as a pharmacological target for myocardial I/R injury.

## Introduction

Myocardial ischemia/reperfusion (I/R) injury is a critical clinical scenario that arises during reperfusion therapy for acute myocardial infarction (AMI) 1, 2. Despite extensive research, effective pharmacological interventions to mitigate myocardial I/R injury have remained elusive 3. Increasing irreversible cardiomyocyte death and aggravating myocardial infarction are the most serious consequences of myocardial I/R injury 4. Given the non-regenerative properties of cardiomyocyte 5, inhibiting cell death is crucial to alleviating myocardial I/R injury. Consequently, there is an urgent need to understand the mechanisms underlying myocardial cell death in I/R injury and to identify new potential therapeutic targets.

Ubiquitination is an essential post-translational modification (PTM) that mediates the degradation of ubiquitinated proteins through the ubiquitin-proteasome system 6. As a reversible process, ubiquitination is regulated by both E3 ubiquitin ligases and deubiquitinating enzymes (DUBs) 7. DUBs are essential for maintaining protein stability and functionality, playing significant roles in numerous biological processes and influencing the progression of various diseases 7. Recently, several DUBs, such as USP7 8, OTUD5 9, have been reported to regulate the progression of myocardial I/R injury. These studies suggested that members of DUBs family could serve as a molecular library for identifying new targets for myocardial I/R injury.

JAB1/MPN/Mov34 metalloenzymes (JAMMs) is the only zinc metalloprotease family of DUBs, whereas the other DUB families are cysteine peptidases 10. The unique catalytic mechanism of JAMMs inducing peptide bond cleavage through zinc atoms makes JAMMs a promising target for drug development 10. Through comparing RNA-seq data and functional screening of JAMMs, we identified Myb-like, SWIRM, and MPN domains 1 protein (MYSM1) as a potential regulator of myocardial I/R injury. MYSM1 was originally discovered as a nuclear chromatin-binding protein and comprises three main domains: SANT, SWIRM, and MPN domain 11. Among them, MPN metalloprotease domain mainly exerts deubiquitinase activity 11. In biological function, MYSM1 emerges as an essential regulator for hematopoiesis and immunity by interacting with specific substrates 11-13.

The purpose of this study was to clarify the regulatory role and mechanism of MYSM1 in myocardial I/R injury. We demonstrated that the expression of MYSM1 positively correlates with myocardial I/R injury, and furthermore, MYSM1 knockdown in cardiomyocytes significantly protects hearts from I/R injury. Through a comprehensive proteome-wide quantitative analysis, we identified signal transducer and activator of transcription 1 (STAT1) as the direct substrate of MYSM1. Mechanistically, MYSM1 promotes the stability of STAT1 by cleaving polyubiquitin chains from the K379 residue of STAT1. In addition, MYSM1 initiates the expression of necroptosis-related genes by promoting the transcription factor function of STAT1. In conclusion, this study illustrated a MYSM1-STAT1 axis in regulating myocardial I/R injury and identified MYSM1 as a pharmacological target for myocardial I/R injury.

## Materials and Methods

### Animal experiments

The whole-body *Mysm1*-knockdown mice (*Mysm1*^-/+^, KD), *Mysm1*-knockout mice (*Mysm1*^-/-^, KO, Strain NO. T058305) with a C57BL/6J background, and littermate wild-type (WT) mice were obtained from Gempharmatech. *Mysm1*^-/-^mice exhibit partial embryonic lethality 11, growth retardation, as well as abnormalities in the hind limb and tail morphology 12, while *Mysm1*^-/+^ mice show no phenotypic abnormalities. The adeno-associated virus serotype 9 (AAV-9), carrying a *cTNT* promoter driving short hairpin RNA (shRNA) targeting *Mysm1* gene sequence (CAGTGATGAGAACAGAGCTATCATT) was constructed by Shanghai Genechem Co. The *cTNT* AAV9-shMYSM1 (total dose 2×10^11^ vg/mouse) or negative control RNA (shNC) was administered to mice via tail vein injection. For the shRNA control, a scrambled sequence (TTCTCCGAACGTGTCACGT) was utilized for the generation of AAV9-shNC. After 4 weeks, the mice were subjected to myocardial I/R injury model.

For the myocardial I/R injury model, the left anterior descending (LAD) coronary artery was ligated and the animals were anaesthetized using 2% isoflurane. After opening the thorax at the fourth intercostal space of the left midclavicular line, the LAD artery was ligated for 30 min using 7-0 silk sutures. The presence of myocardial blanching in the perfusion bed confirmed complete occlusion of the vessel. The ligature was then untied, and reperfusion was initiated for either 4 hours (acute I/R injury) or 2 weeks (chronic I/R injury). After 2 weeks of reperfusion, cardiac function was assessed using transthoracic noninvasive echocardiography with a Vevo 3100 system equipped with an MS-550D ultrasound transducer (Fujifilm Visual Sonics). Isoflurane was used as an anesthetic during the echocardiography. All measurements derived from echocardiography were obtained by averaging the readings from three consecutive cardiac cycles. Subsequently, the mice were humanely euthanized under sodium pentobarbital anesthesia. Finally, blood and heart tissues were collected and stored for further analysis. Collected blood samples were transferred into EDTA-coated tubes and subsequently analyzed using an automated hematology analyzer (88 μL per sample, XN-1000, Sysmex). All animal care and experimental procedures strictly adhered to the Guide for the Care and Use of Laboratory Animals (US National Institutes of Health) and were approved by the ethics committee of the Second Affiliated Hospital of Jiaxing University (approval document no. JUMC2023-102).

### Cell culture, transfection and viability

As previously described, neonatal rat primary cardiomyocytes (NRPCs) were isolated from the ventricles of neonatal Sprague-Dawley rats 14. The HL-1 and NIH/3T3 cells were obtained from the Shanghai Institute of Biochemistry and Cell Biology. HL-1 cells were cultured at 37°C under 5% CO_2_ in Claycomb medium (51800C, Sigma) supplemented with 10% fetal bovine serum (FBS), 1% penicillin/streptomycin, 1% L-glutamine and Norepinephrine. NIH/3T3 cells were cultured at 37°C under 5% CO_2_ in Dulbecco's modified eagle medium (DMEM, Solarbio) supplemented with 10% FBS and 1% penicillin/streptomycin. For the hypoxia/reoxygenation (H/R) model, oxygen-serum deprivation injury was induced by placing cells in a controlled hypoxia incubator (5% CO_2_, 95% N_2_) without serum medium for 4 h. Subsequently, the medium was replaced with normal culture media, and the cells were reoxygenated under normoxic conditions (21% O_2_, 74% N_2_, and 5% CO_2_) for 6 h 15.

Overexpression of MYSM1, STAT1, and other proteins in HL-1 or NIH/3T3 cells was achieved using plasmids (Flag-MYSM1-WT/△MPN/△SWIRM/△SANT, Flag-BRCC3, Flag-COPS5, Flag-PSMD14, Flag-MPND, Flag-STAMBP, Flag-STAMBPL1, Flag-COPS6, Flag-PSMD7, Flag-EIF3H, Flag-EIF3F, Flag-CEBPB, Myc-STAT1-WT/K379R, and HA-Ub/K48/K63) provided by Genechem. Lipofectamine 3000 reagent (L3000-150, Thermo Fisher Scientific) was used to transfect the overexpression plasmids (1 μg plasmid/1 million cells) into the cells. MYSM1 small interfering RNA was constructed (siMYSM1: GCCAGACAAUACUUCAGAATT, Gene Pharma Co. Ltd) to silence MYSM1 expression. The transfection agent Lipofectamine 2000 (11668-019, Thermo Fisher Scientific) was used to transfect siMYSM1 into NRPCs. The knockdown of MYSM1 and STAT1 in HL-1 cells was accomplished using lentiviral-based specific shRNA, constructed and purified by Hanbio Tech. The sequences of the shRNA were shown in **Table S1**. HL-1 cells were infected with the indicated lentiviruses, followed by selection with puromycin (10 μg/mL) for 2 weeks. Single stable cell lines were then generated.

### Interactomics and ubiquitinomics analysis

Interactomics analysis: to investigate MYSM1-binding proteins in cells, HL-1 cells were transfected with Flag-MYSM1 or empty vehicle (EV) plasmids. Cells were harvested using lysis buffer and incubated with anti-Flag-beads (B26102, Bimake) in 4 ℃ overnight. After five washes, the precipitated protein mixtures were added to SDT lysate (4% SDS, 100 mM DTT, 100 mM TrisHCl) for enzymatic hydrolysis and subsequently desalted using C18 Stage Tip. The samples were then subjected to liquid chromatography-tandem mass spectrometry (LC-MS/MS) analysis (Thermo Scientific). The MS data were analyzed using MaxQuant software version 2.0.1.0. The calculation of the score value is embedded within MaxQuant. The data were searched against the UniProt reference proteome Mus musculus database (55,286 total entries). Trypsin was selected as the digestion enzyme, allowing for a maximum of two missed cleavages. The mass tolerance was set to 4.5 ppm for precursor ions and 20 ppm for fragment ions. Carbamidomethylation of cysteines was specified as a fixed modification, while acetylation of protein N-termini and oxidation of methionine were set as variable modifications. The database search results were filtered and exported with a false discovery rate (FDR) of <1% at both the peptide-spectrum match and protein levels.

Ubiquitinomics analysis: HL-1 cells were transfected with Flag-MYSM1 or EV plasmids. Following digestion, the peptides obtained for ubiquitin peptide enrichment were dissolved in NETN buffer and incubated with anti-diglycine remnant (K-ε-GG, 5562, CST) pan antibody beads with gentle shaking (4 °C, overnight). The precipitated peptide mixtures were then subjected to LC-MS/MS analysis. The MS data were analyzed using Proteome Discoverer 2.4. The data were searched against the UniProt Mus musculus database (88,473 total entries). Tryptic cleavage specificity was applied, with variable modifications including oxidation (M), acetylation (protein N-terminus), deamidation (NQ), and Glygly (K), and fixed carbamidomethyl cysteine modification. Peptides were required to have a minimum length of 6 amino acids, and each protein had to be identified by at least one unique peptide. For peptide and protein identification, the FDR was set to 1%. Site quantitation analysis was filtered to include only those phosphorylation sites with a localization probability of ≥0.75, as determined by the algorithm. Label-free quantification was performed using intensity-based methods, and the quantitative site ratios were weighted and normalized by the median ratio. The proteomics raw data have been uploaded to the ProteomeXchange dataset via the PRIDE repository (Accession: PXD058830).

### RT-qPCR

Trizol reagent (15596018CN, Invitrogen) was used to isolate the total RNA from HL-1 cells and heart tissues. After purifying the RNA, the Prime Script RT Master Mix reagent (RR036A, Takara) was used to create the cDNA. Then, the cDNA was used for quantitative RT-PCR by using SYBR Green^TM^ Premix Ex Taq^TM^ II (RR820A, Takara). The amount of each gene was determined and normalized to the amount of *β-actin*. Primer sequences were detailed in **Table S2**.

### CUT&Tag assays

HL-1 cells were transfected with Flag-MYSM1 expressing plasmids (oeMYSM1) or EV plasmids before H/R treatment. The CUT&Tag assay was conducted following the manufacturer's protocol with the NovoNGS CUT&Tag 4.0 HighSensitivity Kit (Novoprotein). In brief, NovoNGS ConA Beads were washed with ConA Binding Buffer. 1x10^5^ cells were prepared and immobilized on concanavalin A beads. Cells with beads were incubated overnight at 4 °C with STAT1 antibody (1:50). Subsequently, the goat anti-rabbit IgG secondary antibody (1:100, N269-01A, Novoprotein) was added into the sample at room temperature for 1.5 h. After removing the unbound secondary antibody, cells were incubated with NovoNGS ChiTag (pAG-Transposome) for 1 h at room temperature. The samples were washed with ChiTag Buffer and then subjected to tagmentation using Tagmentation Buffer. Reactions were stopped by the addition of 5 μL Stop Buffer and 1 μL Proteinase K to each sample and incubated at 55 °C for 10 min. DNA was isolated by NovoNGS DNA Clean Beads and dissolved in TE-RA-Buffer. For high-throughput sequencing and qPCR assays, DNA was amplified with N5 and N7 primers and purified with NovoNGS DNA Clean Beads (Primer sequences were listed in **Table S3**).

### Statistical analysis

Data from both *in vitro* and *in vivo* experiments were expressed as mean ± standard error of the mean (SEM). An unpaired Student's t-test was performed to compare two samples, while ANOVA followed by Bonferroni's Tukey's post hoc test was used to compare multiple samples (GraphPad Pro Prism 8.0). Statistical significance was defined as *P* < 0.05*.*

An extended Materials and Methods section is available in the *Supplementary Information*.

## Results

### The expression of MYSM1 is up-regulated in myocardial I/R injury

**Figure 1:**
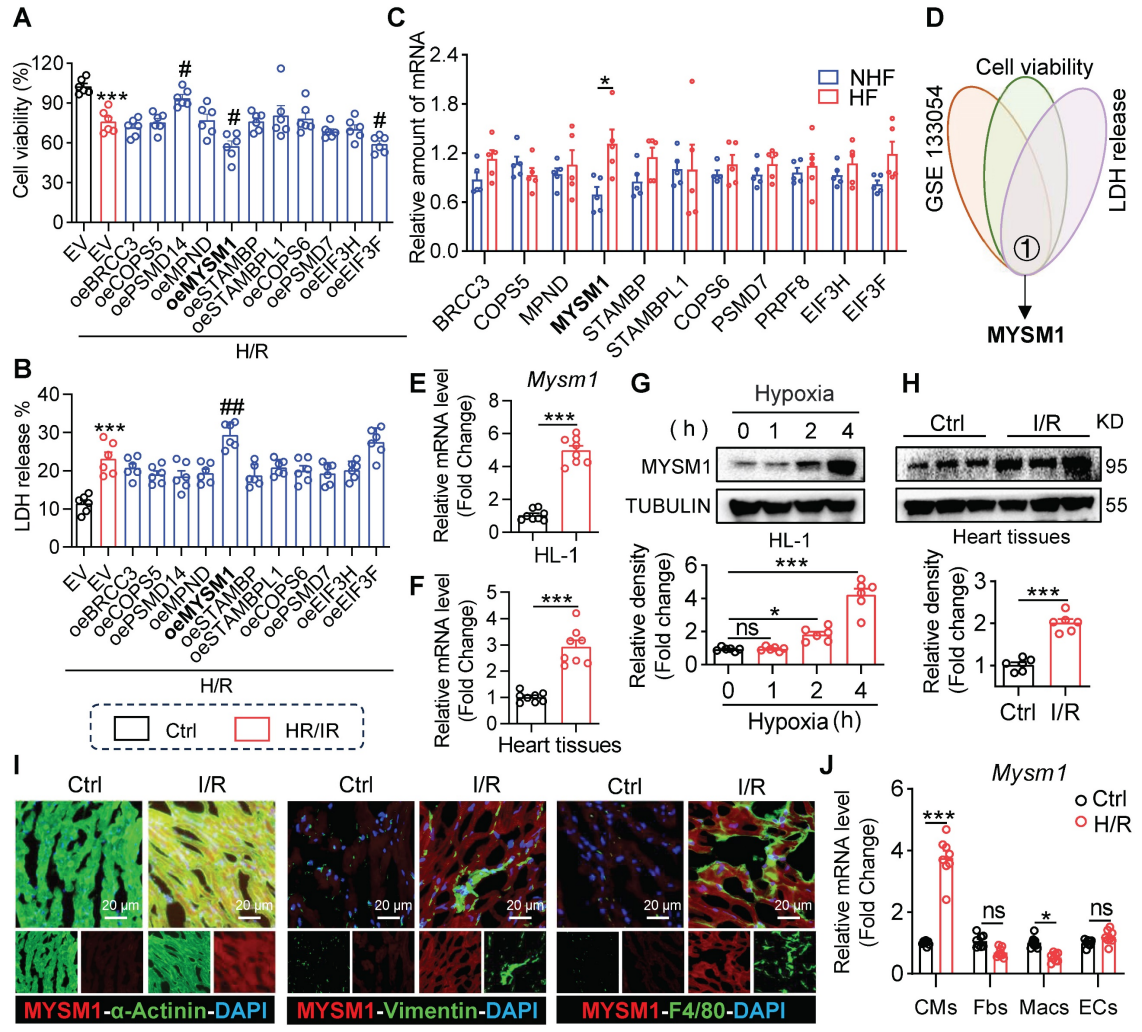
** The expression of MYSM1 is up-regulated in myocardial I/R injury. A-B.** HL-1 cells were transfected with expressing plasmids of JAMMs family (e.g., oeMYSM1) or empty vehicle (EV) before H/R injury (4 h hypoxia, 6 h reoxygenation; n = 6; *, vs. Ctrl; #, vs. EV+H/R; # *P <* 0.05, ## *P <* 0.01, *** *P <* 0.001). (**A**) Cell viability of each group. (**B**) Bar graph showing LDH levels. **C.** The expression levels of JAMMs family genes in mRNA sequencing of human heart tissue (GSE133054, HF=heart failure, NHF=non-heart failure, n = 5). **D.** Comprehensive combined RNA-seq dataset and function screens of JAMMs family for identification of MYSM1 as a regulator in I/R injury. **E-F.** MYSM1 mRNA levels in H/R-induced HL-1 cells (**E**) or myocardial I/R injury (**F**) were detected by RT-qPCR (n = 8). **G-H.** The protein levels of MYSM1 in H/R-induced HL-1 cells (**G**) or myocardial I/R injury (**H**) were examined by western blotting analysis (n = 6). **I.** Representative images of immunofluorescence staining for MYSM1(red), α-actin (green), vimentin (green), or F4/80 (green) in heart sections from mice with I/R injury. Colocalization regions are shown in yellow in merged images. **J.** MYSM1 mRNA of cardiomyocytes (CMs), fibroblasts (Fbs), macrophages (Macs), and endothelial cells (ECs) were detected by RT-qPCR in H/R injury (n = 8). (ns = no significance, * *P <* 0.05, ** *P <* 0.01, *** *P <* 0.001).

To determine the association of JAMMs family genes with myocardial I/R injury, we overexpressed JAMMs family in HL-1 cells (**Figure S1A**) and observed that MYSM1 overexpression resulted in decreased cell viability (**Figure 1A**) and increased LDH release (**Figure 1B**) under H/R treatment. These assays also showed that EIF3F reduced the cell viability and PSMD14 improved cell viability under H/R treatment (**Figure 1A**). Then, we analyzed the expression profiles of JAMMs family genes in human heart tissue using mRNA sequencing (GSE133054, **Figure 1C**). Within this dataset, the mRNA expression of MYSM1 was elevated in heart failure (HF) group compared to the control normal heart (NHF) group. Then By comparing the RNA-seq dataset and functional screening of JAMMs family, we considered MYSM1 as a potential regulator of myocardial I/R injury (**Figure 1D**).

Next, we verified the elevated mRNA levels of MYSM1 in H/R-induced HL-1 cells (**Figure 1E**) and myocardial I/R injury (**Figure 1F**). Similarly, the up-regulations of MYSM1 protein were observed in H/R-induced HL-1 cells (**Figure 1G**) and myocardial I/R injury (**Figure 1H**). Further, we investigated the transcriptional activation of MYSM1 by PROMO database (**Table S4**) and CHIP atlas database (**Table S5**). Through a comparison of PROMO and CHIP atlas, we identified CEBPB as the potential transcription factor of MYSM1 (**Figure S1B**). CEBPB, a transcription factor belonging to the CCAAT-binding family, is predominantly expressed in cardiac tissue and plays a crucial role in the pathogenesis of various cardiovascular diseases, by modulating the transcriptional activity of numerous downstream genes 16, 17. We then examined the effects of CEBPB on MYSM1 expression. As shown in **Figure S1C-D**, both mRNA and protein levels of MYSM1 were elevated in HL-1 cells overexpressing CEBPB, suggesting that CEBPB may function as a cardiac transcription regulator promoting MYSM1's transcriptional activity. To investigate the cellular distribution of MYSM1 in hearts, double immunofluorescence staining was performed and revealed that MYSM1 was predominantly increased in α-actinin^+^ cardiomyocytes, but not in vimentin^+^ fibroblasts and F4/80^+^ infiltrated macrophages (**Figure 1I**). The up-regulation of MYSM1 mRNA level was also observed specifically in cardiomyocytes rather than non-cardiomyocytes (**Figure 1J**). Therefore, MYSM1 was identified as a potential regulator in myocardial I/R injury.

### MYSM1 knockdown mice protect against myocardial I/R injury *in vivo*

**Figure 2:**
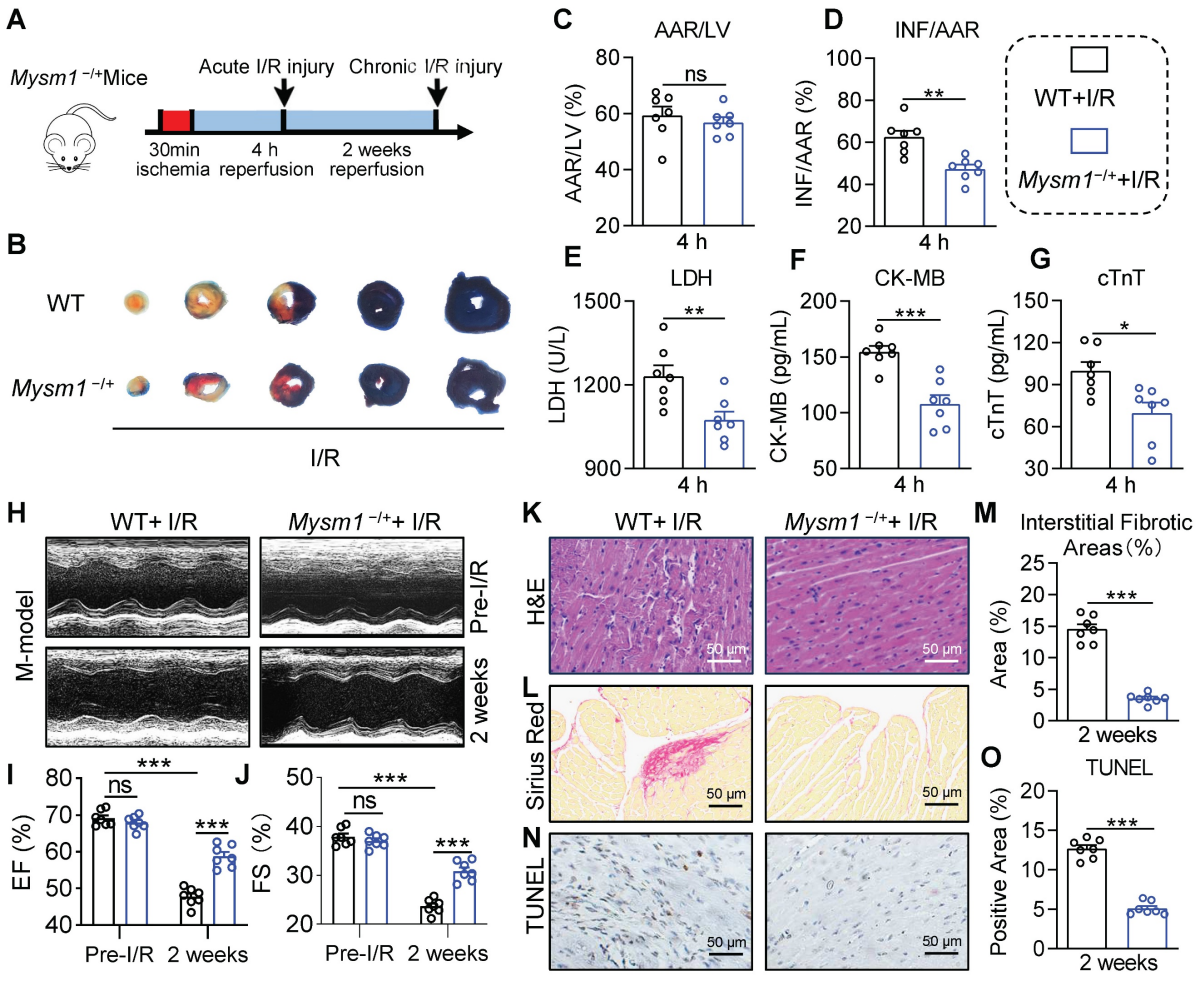
** MYSM1 knockdown mice protect against myocardial I/R injury *in vivo*. A.** The workflow of the *Mysm1*^-/+^mice subjected to acute I/R injury (30 min of ischemia and 4 h of reperfusion) or chronic I/R injury (30 min of ischemia and 2 weeks of reperfusion). **B-D.** The Myocardial infarction size determined by Evans blue (EB)/triphenyl tetrazolium chloride (TTC) double-staining and quantitative data for area at risk (AAR, **C**) and infarct size (INF, **D**, n = 7). **E-G.** The levels of serum lactic dehydrogenase (LDH, **E**), creatine kinase-MB (CK-MB, **F**), and cardiac troponin T (cTnT, **G**) following acute myocardial I/R injury (n = 7). **H.** Mice M-mode echocardiography images from each group. **I-J.** Relative levels of ejection fraction (EF%, **I**) and fractional shortening (FS%, **J**) in mice (n = 7). **K.** Images of longitudinal heart tissue sections stained with H&E. **L, M.** Images of Sirius red staining for fibrosis tissue and bar graph showing the quantification of interstitial fibrosis (%) (n = 7). **N, O.** Apoptotic DNA fragmentation was assessed by TUNEL staining (brown: TUNEL-positive nuclei), accompanied by a bar graph illustrating the quantification (n = 7). (ns = no significance, * *P <* 0.05, ** *P <* 0.01, *** *P <* 0.001).

To investigate the function of MYSM1 in myocardial I/R injury, we generated the *Mysm1*-deficiency mice (**Figure S2A**). Previous studies have reported that MYSM1 knockout mice exhibit partial embryonic lethality, complex hematopoietic immune phenotypes, growth retardation, reduced lifespan, and abnormalities in the tail morphology 18, 19. Conversely, heterozygous *Mysm1*^-/+^ mice (**Figure S2B-C**) did not exhibit abnormal blood parameters (**Figure S2D-G**). Hence, *Mysm1*^-/+^ mice were chosen for further investigation. We used an acute myocardial I/R model with 30 min of ischemia followed by 4 h of reperfusion (**Figure 2A**). Infarct size was assessed using EB/TTC staining. As shown in** Figure 2B-D**, *Mysm1*^-/+^ mice had reduced myocardial infarct area compared to WT mice following I/R injury. Additionally, *Mysm1*^-/+^ mice displayed a significant decrease in serum cardiac injury markers, including LDH, CK-MB, and cTnT, compared to WT mice after I/R injury (**Figure 2E-G**). As shown in **Figure S2H-J**, MYSM1 knockdown also ameliorated cardiac dysfunction in mice subjected to acute myocardial I/R injury.

Subsequently, we used a chronic myocardial I/R model with 30 min of ischemia followed by 2 weeks of reperfusion (**Figure 2A**). Compared with the WT mice, noninvasive echocardiography revealed that MYSM1 knockdown improved cardiac function in I/R-treated mice, evidenced by increased ejection fraction (EF%) and fractional shortening (FS%) (**Figure 2H-J and Table S6**). H&E staining of heart tissues demonstrated that MYSM1 knockdown prevented I/R-induced impairment of myocardial morphology (**Figure 2K**). Sirius Red staining of heart tissues indicated that MYSM1 knockdown attenuated excessive fibrosis in myocardial I/R injury (**Figure 2L-M**). Furthermore, TUNEL staining showed a marked reduction in cell death in cardiac tissues of *Mysm1*^-/+^ mice subjected to I/R injury (**Figure 2N-O**). *Mysm1*^-/+^ mice also resulted in a reduction of myocardial infarct area (**Figure S2K-L**) and serum cardiac injury markers (**Figure S2M-O**) compared to WT mice following chronic I/R injury. These findings suggested that MYSM1 knockdown protects against myocardial I/R injury in *vivo*.

### Cardiomyocyte-specific knockdown of MYSM1 protects against myocardial I/R injury

**Figure 3:**
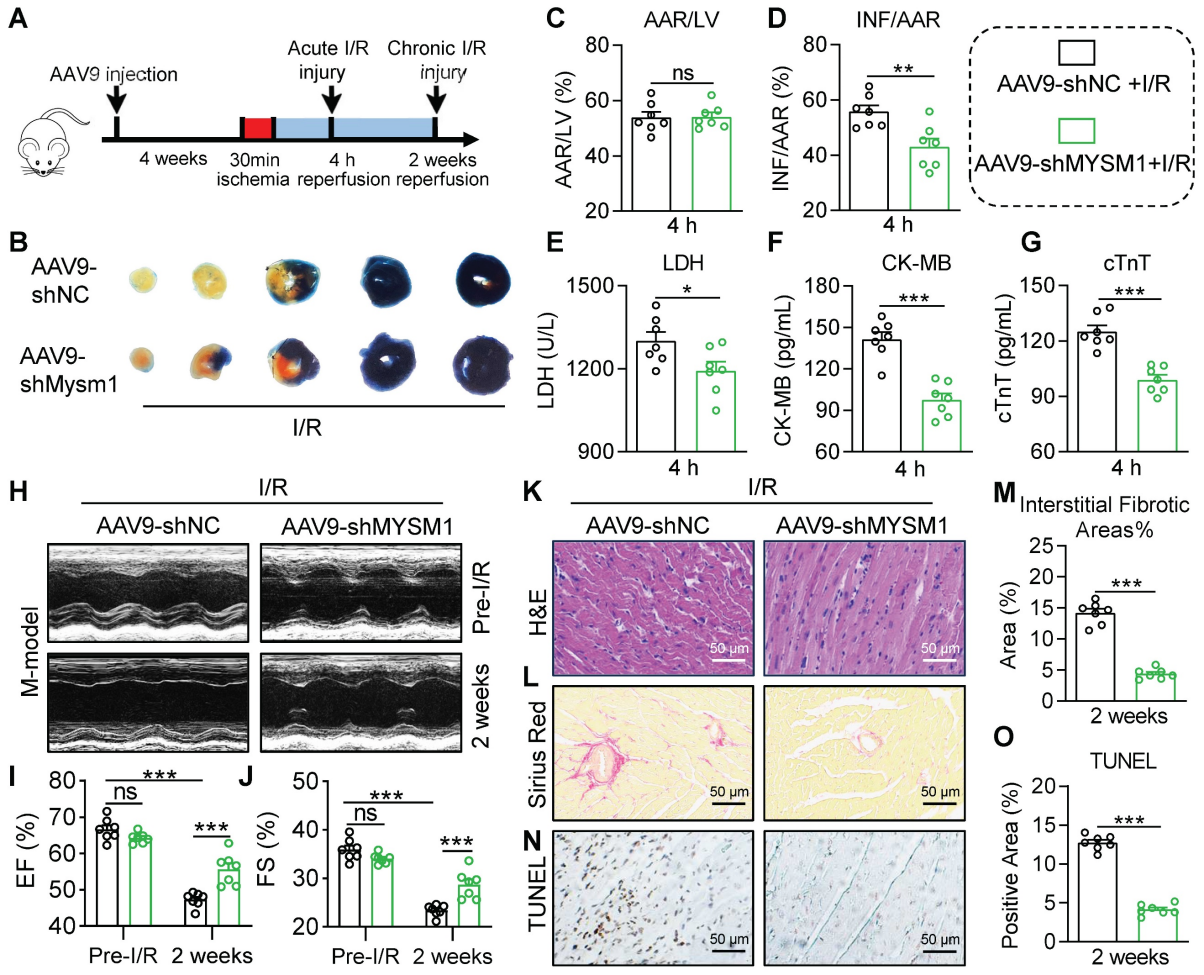
**Cardiomyocyte-specific knockdown of MYSM1 protects against myocardial I/R injury. A.** AAV9s (shMYSM1, or shNC) were injected to mice by the tail vein (total dose 2×10^11^ vg). After 4 weeks, the mice were subjected to myocardial I/R injury model. **B-D.** Myocardial infarction size determined by EB/TTC double-staining and quantitative data for AAR (**C**) and INF (**D**) (n = 7). **E-G.** The levels of serum LDH (**E**), CK-MB (**F**), and cTnT (**G**) following acute myocardial I/R (n = 7). **H.** Mice M-mode echocardiography images from each group. **I, J.** Relative levels of EF% (**I**) and FS% (**J**) in mice (n = 7). **K.** Images of longitudinal heart tissue sections stained with H&E. **L, M.** Images of Sirius red staining for fibrosis tissue and bar graph showing the quantification of interstitial fibrosis (%) (n = 7). **N, O.** Apoptotic DNA fragmentation was assessed by TUNEL staining (brown: TUNEL-positive nuclei), accompanied by a bar graph illustrating the quantification (n = 7). (ns = no significance, * *P <* 0.05, ** *P <* 0.01, *** *P <* 0.001).

To specifically block cardiomyocyte MYSM1 expression *in vivo*, AAV-9 vector carrying a *cTNT* promoter driving MYSM1 short-hairpin RNA (shMYSM1) was prepared. The efficacy of shMYSM1 in suppressing MYSM1 expression was confirmed in heart tissues (**Figure S3A**). Injection of AAV9-*cTNT* did not affect blood parameters in mice (**Figure S3B-E**). We employed a mouse model of acute cardiac I/R injury as described above (**Figure 3A**). EB/TTC staining was used to determine the infarct size. As depicted in** Figure 3B-D**, AAV9-shMYSM1 mice showed an inhibitory effect on the I/R-induced myocardial infarct area compared to AAV9-shNC mice. Additionally, the AAV9-shMYSM1 mice exhibited a significant decrease in serum cardiac injury markers, including LDH, CK-MB and cTnT, compared to the AAV9-shNC mice after I/R injury (**Figure 3E-G**). As shown in **Figure S3F-H**, cardiomyocyte-specific knockdown of MYSM1 also ameliorated cardiac dysfunction in mice subjected to acute myocardial I/R injury.

In the mouse chronic cardiac I/R model (**Figure 3A**), noninvasive echocardiography showed that the AAV9-shMYSM1 mice ameliorated I/R-induced cardiac dysfunction compared with the AAV9-shNC mice, manifested by increased EF% and FS% (**Figure 3H-J and Table S7**). H&E staining of heart tissues demonstrated that the AAV9-shMYSM1 mice mitigated I/R-induced impairment of myocardial morphology (**Figure 3K**). Sirius Red staining of heart tissues indicated that AAV9-shMYSM1 mice ameliorated excessive fibrosis induced by I/R injury. (**Figure 3L-M**). In addition, TUNEL staining revealed that cardiac tissues of AAV9-shMYSM1 mice had significantly reduced cell death after I/R injury (**Figure 3N-O**). As shown in **Figure S3I-M**, cardiomyocyte-specific knockdown of MYSM1 also resulted in a reduction of myocardial infarct area and serum cardiac injury markers following chronic I/R injury. These results substantiated the protective effects of cardiomyocyte-specific MYSM1 knockdown against myocardial I/R injury.

### MYSM1 promotes H/R-induced cardiomyocyte injury *in vitro*

**Figure 4:**
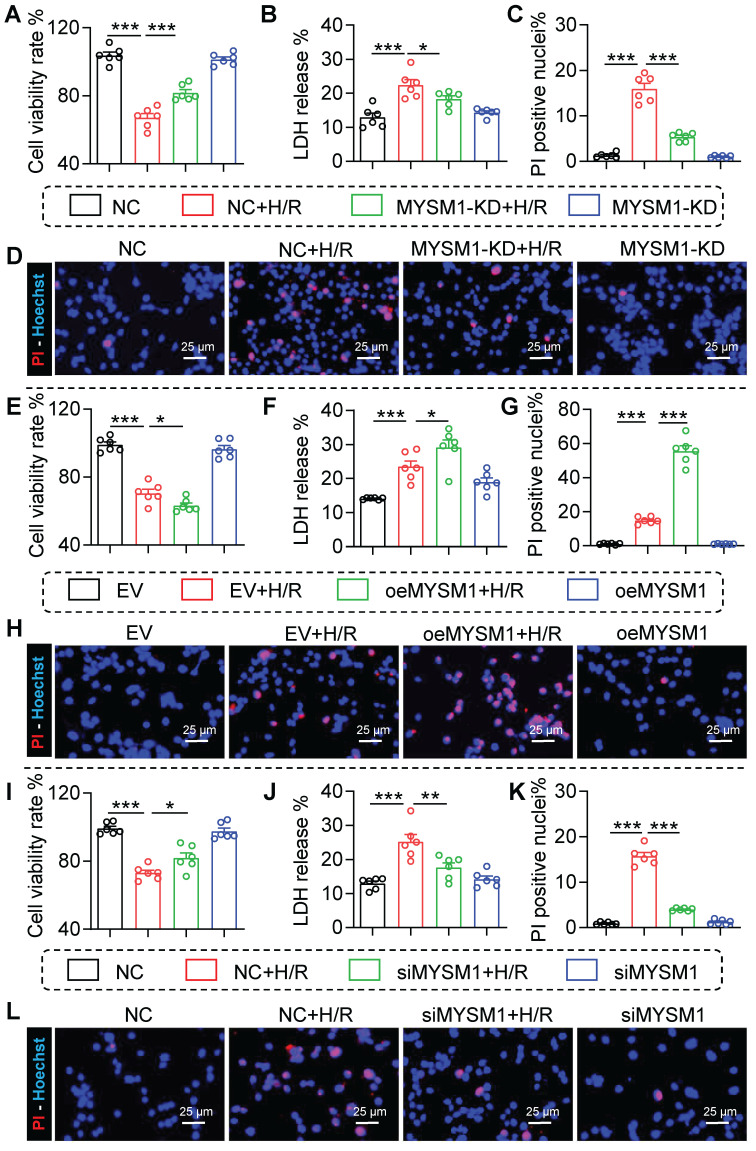
** The loss-of-function and gain-of-function of MYSM1 were evaluated in H/R-treated cardiomyocyte. A-D.** MYSM1 knockdown (KD) HL-1 cells were constructed via shRNA (shMYSM1) before H/R injury (4 h of hypoxia and 6 h of reoxygenation).** (A)** Cell viability was assessed using the CCK8 assay (n = 6). **(B)** Bar graph showing the levels of LDH (n = 6). (**C, D**) The effect of MYSM1 knockdown on H/R-induced apoptosis in HL-1 determined by PI staining. Representative images for PI staining are shown (**D**), with quantitative columns for PI-positive cells (**C**, n = 6). **E-H.** MYSM1 was overexpressed by transfecting HL-1 cells with Flag-MYSM1 (oeMYSM1) before H/R injury. (**E**) Cell viability was assessed using the CCK8 assay (n = 6). (**F**) Bar graph showing the levels of LDH (n = 6). (**G-H**) The effect of oeMYSM1 on H/R-induced apoptosis in HL-1 determined by PI staining. Representative images for PI staining are shown (**H**), with quantitative columns for PI-positive cells (**G**, n = 6). **I-L.** MYSM1 was knocked down by siRNA transfection (siMYSM1) in NRPCs before H/R injury. (**I**) Cell viability was assessed using the CCK8 assay (n = 6). (**J**) Bar graph showing the levels of LDH (n = 6). (**K-L**) The effect of siMYSM1 on H/R-induced apoptosis in NRPCs was determined by PI staining. Representative images for PI staining are shown (**L**), with quantitative columns for PI-positive cells (**K**, n = 6). (* *P <* 0.05, ** *P <* 0.01, *** *P <* 0.001).

Our previous data suggested that MYSM1 knockdown protected against myocardial I/R injury, with MYSM1 expression mainly upregulated in I/R-stimulated cardiomyocytes. To further explore the role of MYSM1 *in vitro*, the MYSM1-knockdown (MYSM1-KD) HL-1 cells were constructed via shRNA (shMYSM1, **Figure S4A**). Notably, MYSM1 knockdown significantly enhanced cell viability (**Figure 4A**) and inhibited the activity of LDH in the cellular supernatant (**Figure 4B**) under H/R injury. Additionally, MYSM1 knockdown reduced the quantity of Propidium Iodide (PI)-positive nuclei during H/R injury (**Figure 4C-D**).

In a manner analogous to the assessment of loss-of-function, the gain-of-function of MYSM1 was evaluated by transfecting HL-1 cells with oeMYSM1 (**Figure S4B**). Our study indicated that upregulation of MYSM1 led to decreased cellular viability (**Figure 4E**) and elevated LDH release during H/R injury (**Figure 4F**). We further found that upregulation of MYSM1 increased the quantity of PI-positive nuclei following H/R injury (**Figure 4G-H**).

As previously described, NRPCs were isolated from the left ventricles of neonatal SD rats 14, and MYSM1 was silenced by siRNA transfection (siMYSM1, **Figure S4C**). As shown in **Figure 4I**-**J**, MYSM1 silencing markedly increased cell viability (**Figure 4I**) and suppressed the LDH release (**Figure 4J**) during H/R injury. Furthermore, MYSM1 silencing decreased PI-positive nuclei in H/R injury (**Figure 4K-L**). These results validated that MYSM1 contributes to H/R-induced cardiomyocyte injury *in vitro*.

### STAT1 was identified as the crucial substrate protein of MYSM1

**Figure 5:**
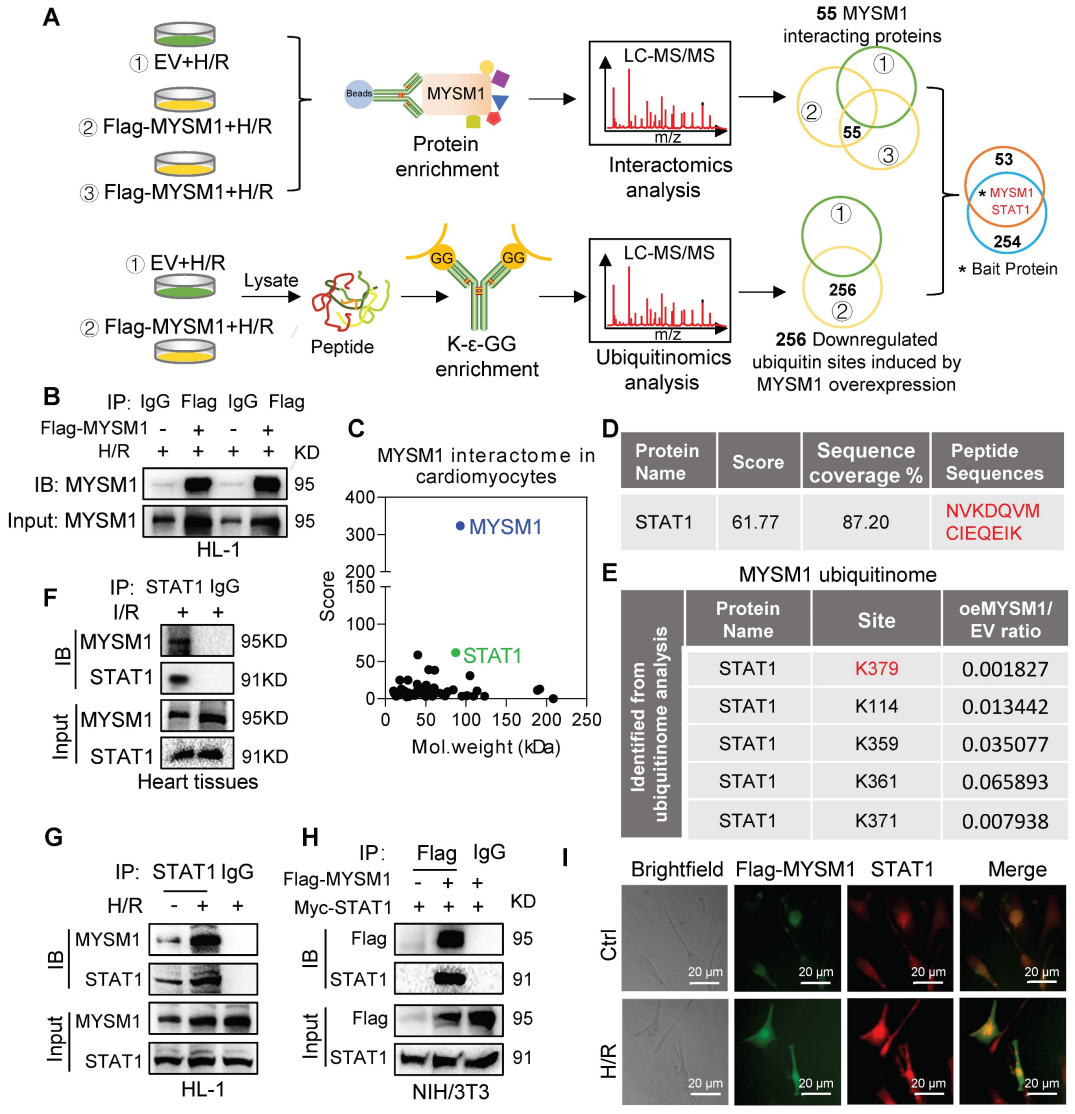
** Identification of STAT1 as a potential substrate of MYSM1. A.** Schematic of a comprehensive combined interactomics and ubiquitinomics analysis for MYSM1 substrate screening. HL-1 cells were transfected with Flag-MYSM1 or EV plasmid and cultured for 24 h. For interactomics analysis, cells were harvested using lysis buffer and added with anti-Flag-beads at 4℃ overnight. Then samples were subjected to LC-MS/MS analysis. For ubiquitinomics analysis, the peptides digested from protein were subjected to ubiquitin peptide enrichment and then analyzed by LC-MS/MS. **B.** Representative western blotting results of Co-IP samples. HL-1 cells were transfected with Flag-MYSM1 or EV plasmid before H/R injury. The results showed transfection of the Flag-MYSM1 plasmid induced an increase in MYSM1 levels in IP products. **C.** Two-dimensional plot revealing the score of proteins on the y-axis and the molecular mass of proteins on the x-axis. **D.** The binding peptide sequences and score between MYSM1 and STAT1 analyzed from interactomics analysis. **E.** Ubiquitinomics analysis revealed that downregulated ubiquitination lysine residues of the potential substrate (STAT1). **F.** Co-IP of STAT1 in myocardial I/R injury. Endogenous MYSM1 was immunoprecipitated by anti-STAT1 antibody. **G.** Co-IP of STAT1 in H/R-induced HL-1 cells. Endogenous MYSM1 was immunoprecipitated by anti-STAT1 antibody. **H.** Co-IP of Flag-MYSM1 in NIH/3T3 cells overexpressed with Flag-MYSM1 and Myc-STAT1. Exogenous Flag-MYSM1 was immunoprecipitated by anti-Flag antibody. Western blot detected the protein levels of Flag-MYSM1 and STAT1. **I.** Colocalization of Flag-MYSM1 (green) and STAT1 (red) in H/R-induced HL-1 cells. Yellow in merged images denotes regions of colocalization.

MYSM1, as a DUB, primarily exerts its biological functions through specific substrate proteins. To further explore potential substrates of MYSM1, we conducted a comprehensive analysis integrating interactomics and ubiquitinomics assays in H/R-stimulated cardiomyocytes. Firstly, we analyzed the binding proteins of MYSM1 by Co-IP with LC-MS/MS (interactomics analysis) in HL-1 cells overexpressing MYSM1 (**Figure 5A upper and Figure 5B**). The results revealed 55 proteins physically interacting with MYSM1 (**Figure 5C**). Then, we enriched ubiquitinated peptides using K-ε-GG and performed LC-MS/MS analysis to identify the deubiquitination substrates of MYSM1 (**Figure 5A below**). A total of 256 modified proteins showed significantly reduced ubiquitination levels in cells overexpressing Flag-MYSM1 (**Figure 5A below**). Finally, by comparing interacting proteins and modified proteins with downregulated ubiquitinated sites, we proposed that STAT1 may be a potential deubiquitination substrate of MYSM1 (**Figure 5A and Figure 5D**). Moreover, ubiquitinomics analysis revealed that several lysine residues of STAT1 were downregulated by the deubiquitination effect of MYSM1 (**Figure 5E**). STAT1 has been reported to promote myocardial I/R injury 20, 21. Therefore, we speculated that MYSM1 may utilize STAT1 as a crucial substrate in the regulation of myocardial I/R injury.

Then we examined the direct interaction between MYSM1 and STAT1. The binding of endogenous MYSM1 and STAT1 was observed in myocardial I/R injury (**Figure 5F**) and H/R-induced HL-1 cells (**Figure 5G**). We also performed a Co-IP assay in NIH/3T3 co-transfecting with Flag-MYSM1 and Myc-STAT1. The results demonstrated that exogenous Flag-MYSM1 and Myc-STAT1 were also efficiently co-immunoprecipitated (**Figure 5H**). Immunofluorescence and colocalization analysis further confirmed a significant proportional colocalization of MYSM1 and STAT1 in H/R-induced HL-1 cells (**Figure 5I**). These findings confirmed a direct interaction between MYSM1 and STAT1, suggesting that STAT1 may be a crucial substrate protein of MYSM1.

### MYSM1 deubiquitinates STAT1 and maintains the stability of STAT1

**Figure 6:**
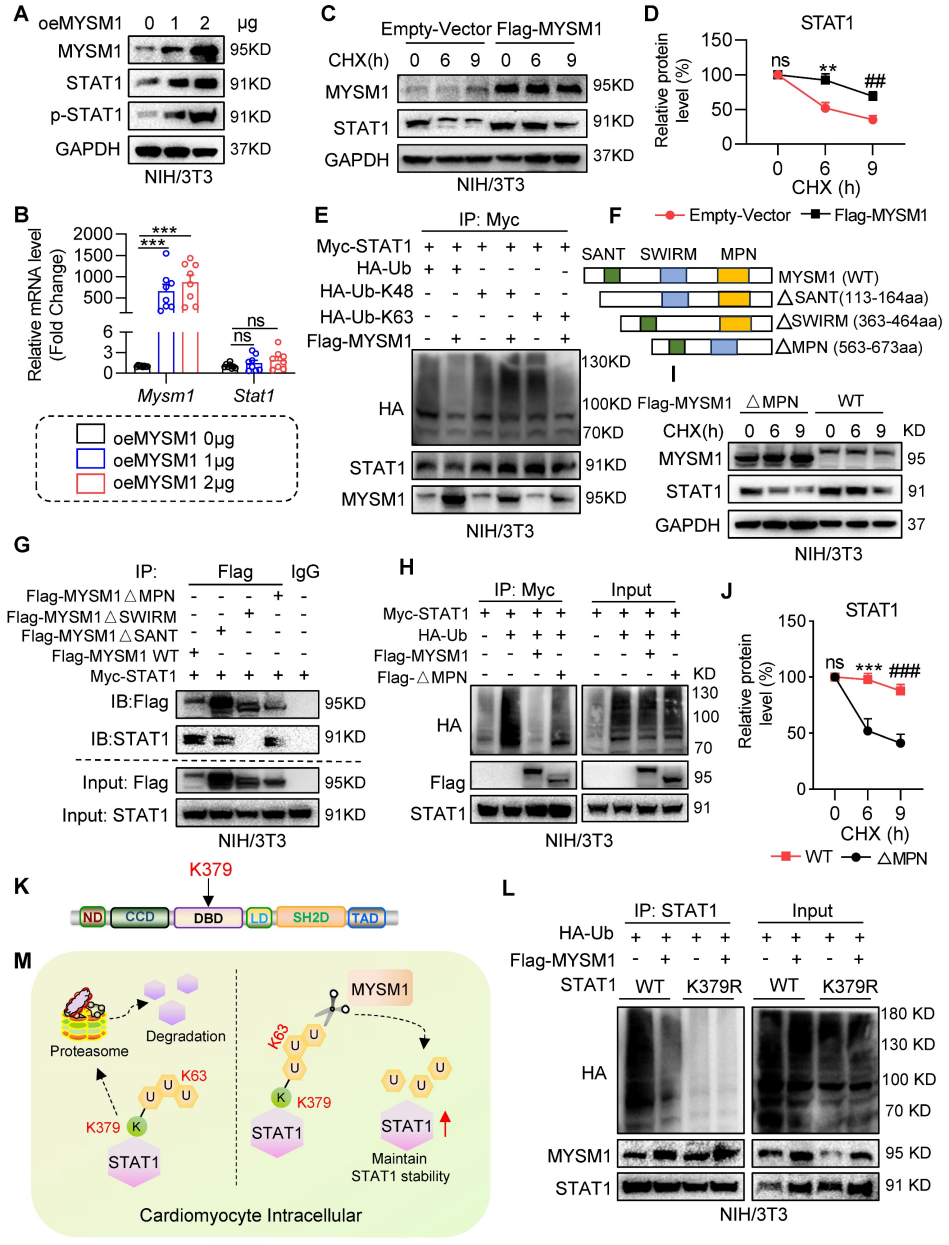
** MYSM1 deubiquitinates STAT1 and maintains the stability of STAT1. A.** NIH/3T3 cells were transfected with Flag-MYSM1 (1 or 2 μg) then analyzed by western blot for MYSM1, STAT1, p-STAT1 (Tyr701), and GAPDH (loading control). **B.** Representative RT-qPCR for *Mysm1* and *Stat1* in NIH/3T3 cells transfected with Flag-MYSM1 or EV plasmid (ns = no significance, *** P < 0.001, n = 7). **C, D.** NIH/3T3 cells were transfected with Flag-MYSM1 and EV plasmid, treated with CHX (25 μg/mL). (**C**) Western blot analysis for MYSM1, STAT1, and GAPDH (loading control). (**D**) Densitometric quantification of STAT1 (ns = no significance vs. Vector-0; *, vs. Vector-6; #, vs. Vector-9; ** *P <* 0.01, ## *P <* 0.01, n = 6). **E.** NIH/3T3 cells were co-transfected with Flag-MYSM1, Myc-STAT1, HA-Ub, HA-K48, and HA-K63, and treated with 10 μM MG132 for 4 h before collection. Cell lysates were treated with anti-Myc antibody. Ubiquitinated STAT1 was detected by western blot. **F.** Schematic of MYSM1 and its deletion mutants, including MYSM1(∆SANT), MYSM1(∆SWIRM), and MYSM1(∆MPN). **G.** NIH/3T3 cells were co-transfected with Myc-STAT1, Flag-MYSM1-WT or ∆MPN, ∆SWIRM and ∆SANT mutants, and treated with 10 μM MG132 for 4 h before collection. Cell lysates were treated with anti-Flag antibody to identify the binding region of MYSM1 regulating deubiquitination of STAT1. **H.** NIH/3T3 cells were co-transfected with Myc-STAT1, HA-Ub, Flag-MYSM1-WT or MPN mutants, and treated with 10 μM MG132 for 4 h before collection. Cell lysates were treated with anti-Myc antibody to identify the active region of MYSM1 regulating deubiquitination of STAT1. **I, J.** NIH/3T3 cells were transfected with Flag-MYSM1-WT or MPN mutants, and subjected to CHX (25 μg/mL). (**I**) Western blotting for MYSM1, STAT1, and GAPDH (loading control). (**J**) Densitometric quantification of STAT1 (ns = no significance vs. WT-0; *, vs. WT-6; #, vs. WT-9; *** P < 0.001, ### *P <* 0.001, n = 6). **K.** Schematic of the STAT1 ubiquitinated lysine residue variant (K379R) construct. **L.** NIH/3T3 cells were transfected with Flag-MYSM1, HA-Ub, and Myc-STAT1-WT/ STAT1-K379R, and treated with 10 μM MG132 for 4 h before collection. Cell lysates were treated with anti-STAT1 antibody to identify the residue of MYSM1 regulating deubiquitination of STAT1. **M.** Scheme of the mechanism of STAT1 deubiquitination by MYSM1.

Initially, we investigated whether MYSM1 regulates STAT1 stability. We transfected NIH/3T3 cells with oeMYSM1 and found that the protein levels of both STAT1 and p-STAT1 (Tyr701) were increased following overexpression of MYSM1 (**Figure 6A and Figure S5A**). This increase was not due to altered transcription of STAT1 (**Figure 6B**). Next, we treated NIH/3T3 cells with the protein synthesis inhibitor cycloheximide (CHX) and measured the degradation of STAT1. As presented in **Figure 6C-D**, the degradation rate of STAT1 was reduced in Flag-MYSM1 overexpressing NIH/3T3 cells under CHX treatment. MYSM1 was found to enhance the protein stability of STAT1, which correspondingly resulted in an increased phosphorylation level of STAT1. However, it is worth exploring whether MYSM1 modulates STAT1 phosphorylation via receptor tyrosine kinases or phosphatases. Currently, JAK1 and SHP2 are acknowledged as the upstream receptor tyrosine kinase and phosphatase of STAT1, respectively 22, 23. Thus, we examined whether MYSM1 binds to and maintains the protein stability of JAK1 and SHP2. The Co-IP results showed that MYSM1 did not bind to either JAK1 or SHP2 protein (**Figure S5B-C**). Additionally, overexpression of MYSM1 did not affect the protein levels of JAK1, p-JAK1 and SHP2 (**Figure S5D-E**). These results indicate that MYSM1 primarily enhances STAT1 phosphorylation by increasing the stability of the STAT1 protein, rather than through its receptor tyrosine kinases or phosphatases.

We then explored the regulation of MYSM1 on STAT1 ubiquitination. NIH/3T3 cells were co-transfected with Myc-STAT1, Flag-MYSM1 and HA-Ub or its mutants (HA-K48/K63). As shown in **Figure 6E**, oeMYSM1 promoted the deubiquitination of STAT1. It has been reported that MYSM1 removes K63-linked ubiquitination and maintains the protein stability of its substrates 24. Consistently, MYSM1 reduced K63-linked ubiquitination of STAT1 but not K48-linked ubiquitination (**Figure 6E**).

Structurally, MYSM1 consists of the MPN, SANT and SWIM domains 11. To determine the domain required for MYSM1-STAT1 interaction, we co-transfected NIH/3T3 cells with Myc-STAT1, Flag-MYSM1 (MYSM1-WT) or its mutants lacking SANT domain (△SANT), SWIRM domain (△SWIRM), or MPN domain (△MPN) (**Figure 6F**). As depicted in **Figure 6G**, STAT1 interacted with MYSM1-WT, MYSM1-△SANT, and MYSM1-△MPN, but failed to bind to MYSM1-△SWIRM, indicating that MYSM1 binds to STAT1 through the SWIRM domain. The MPN metalloprotease domain contributes to the catalytic domain of MYSM1 11. As expected, MYSM1-△MPN failed to remove ubiquitin molecules from STAT1 (**Figure 6H**). Similarly, the ability of MYSM1-△MPN to maintain the stability of STAT1 protein was also decreased (**Figure 6I-J**). These data indicated that MYSM1 interacts with STAT1 through the SWIRM domain and deubiquitinates STAT1 through the MPN domain.

According to our ubiquitinomics, the K379 residue of STAT1, located in the DNA binding domain (DBD) of STAT1 (**Figure 6K**), was the most significantly reduced deubiquitination residue of STAT1 after MYSM1 overexpression (**Figure 5E**). Thus, we constructed a K379R mutant (mutation of lysine to arginine at Lys-379) of STAT1. The ubiquitination level of STAT1-K379R was significantly lower than that of WT STAT1 (**Figure 6L**). It's worth noting that the polyubiquitination of STAT1-K379R was not further reduced by oeMYSM1. Taken together, these results suggested that MYSM1 promotes STAT1 stability by cleaving the polyubiquitin chains on K379 residue of STAT1 (**Figure 6M**).

### MYSM1 initiates the expression of necroptosis-related genes via promoting the transcription factor function of STAT1

**Figure 7:**
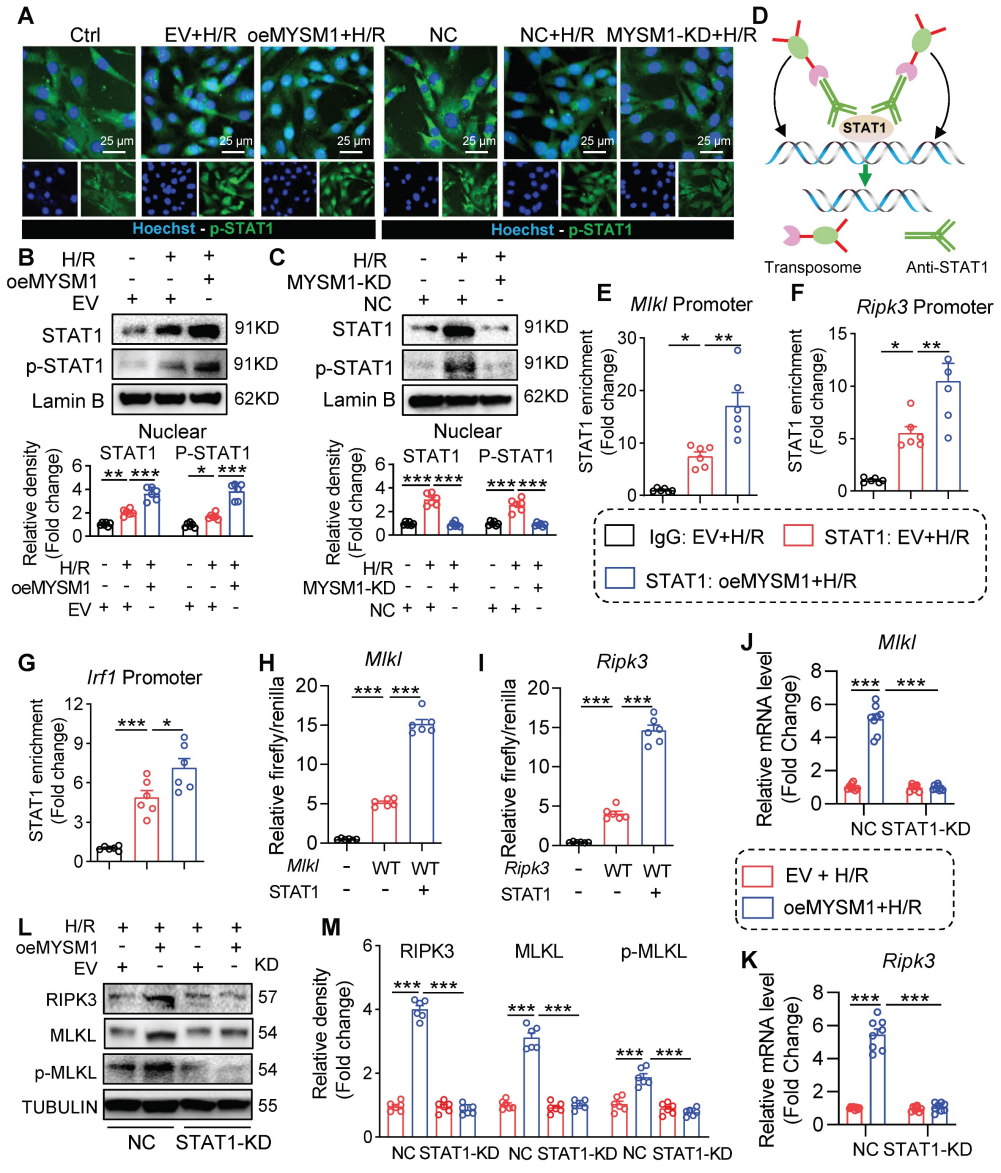
** MYSM1 initiates the expression of necroptosis-related genes via promoting the transcription factor function of STAT1. A-C.** HL-1 cells were transfected with Flag-MYSM1 (oeMYSM1) or EV plasmid. MYSM1 knockdown (KD) HL-1 cells were constructed via shRNA (shMYSM1) before H/R injury (4 h of hypoxia and 6 h of reoxygenation). (**A**) P-STAT1 (green) and nucleus marker Hoechst (blue) were examined under fluorescent microscope. (**B, C**) Representative western blotting analysis of STAT1, p-STAT1 and Lamin B (loading control) (n = 6). **D.** Schematic of CUT&Tag experiments to map the genomic occupancy of STAT1. **E-G.** CUT&Tag-qPCR verified the binding of STAT1 at the promoter regions of *Mlkl* (**E**),* Ripk3* (**F**), and* Irf1* (**G**) (n = 6). **H-I.** Dual luciferase reporter assay detected the luciferase activation driven by *Mlkl* (**H**) or *Ripk3*(**I**) promoter after normalization to renilla luciferase (n = 6). **J-M.** We transfected with oeMYSM1 or EV plasmid in STAT1-KD or NC HL-1 cells before H/R treatment. (**J-K**) RT-qPCR analysis of mRNA level of *Mlkl* (**J**), and *Ripk3* (**K**) (n = 8). (**L**) Western blotting of protein levels of RIPK3, MLKL, p-MLKL, and TUBULIN (loading control) (n = 6). (ns = no significance, * *P <* 0.05, ** *P <* 0.01, *** *P <* 0.001).

STAT1 functions as a transcription factor by undergoing phosphorylation, translocating to the nucleus, and binding to specific promoter regions of target genes 25. Above all, we investigated the impact of MYSM1 on the nuclear translocation of STAT1. As shown in** Figure 7A-B**, upregulation of MYSM1 (oeMYSM1) led to a significant increase in H/R-mediated P-STAT1 nuclear translocation in HL-1 cells. On the contrary, MYSM1 knockdown (MYSM1-KD) resulted in a decrease in the nuclear level of P-STAT1 (**Figure 7A and Figure 7C**). As illustrated in **Figure S6A**, MYSM1 knockdown (*Mysm1^-/+^*) also led to a reduction in the nuclear levels of P-STAT1 *in vivo*.

Subsequently, we examined the genomic occupancy of STAT1 by CUT&Tag assay (**Figure 7D**). STAT1 plays a significant role in programmed cell deaths (PCDs) 26, 27. STAT1-dependent regulation of PCD is largely dependent on a transcriptional mechanism, such as the binding of STAT1 to promoter regions of target genes 28. Therefore, we screened for PCDs-related genes regulated by STAT1 using CUT&Tag sequencing. As shown in **Table S8**, STAT1 peaks were enriched in the promoter regions of several PCDs-related genes, including *Mlkl*, *Ripk3*, *Ripk1*, *Fas*, *Zbp1*, and *Casp3*. Besides, we found that oeMYSM1 only promoted the mRNA expression of necroptosis-related genes (*Mlkl*/*Ripk3*) rather than other PCD-related genes (*Ripk1*/*Fas*/*Zbp1*/*Casp3*) in H/R-induced HL-1 cells (**Figure S6B-G**), indicating that MYSM1-STAT1 axis up-regulated the transcription and expression of necroptosis-related genes. *Mysm1^-/+^* also attenuated the I/R-mediated upregulation of *Mlkl* and *Ripk3* mRNAs in the myocardial infarcted area (**Figure S6H-I**). Through CUT&TAG-qPCR assay, we validated STAT1 binding to the promoter regions of *Mlkl*, *Ripk3*, and *Irf1* (a positive control gene for STAT1), with enhanced binding facilitated by oeMYSM1 (**Figure 7E-G**). Additionally, we cloned the promoter regions of *Mlkl* and* Ripk3* and performed the luciferase promoter assay. As shown in **Figure 7H-I**, STAT1 enhanced the luciferase activity driven by the* Mlkl* and *Ripk3* promoters, indicating that STAT1 binds to the promoter regions of *Mlkl* and *Ripk3* and activates their transcription.

Next, we transfected with oeMYSM1 or EV plasmid in STAT1-knockdown or NC HL-1 cells before H/R treatment. As anticipated, oeMYSM1 markedly up-regulated the mRNA expression of *Mlkl* and *Ripk3* in H/R-induced HL-1 cells (**Figure 7J-K**). Notably, STAT1 knockdown (STAT1-KD, **Figure S6J**) reversed MYSM1-mediated up-regulation of *Mlkl* and *Ripk3* mRNAs (**Figure 7J-K**), confirming that MYSM1 transcriptionally increased the expression of necroptosis-related genes via STAT1. Likewise, oeMYSM1 exacerbated H/R-induced protein expression of RIPK3, MLKL, and p-MLKL, whereas this altered profile was abolished by STAT1-KD (**Figure 7L-M**). In summary, these results showed that MYSM1 initiates the expression of necroptosis-related genes by promoting the transcription factor function of STAT1.

## Discussion

In this study, we demonstrated a positive correlation between MYSM1 expression and myocardial I/R injury. Genetic knockdown of MYSM1 significantly conferred protection against I/R injury in hearts. Correspondingly, AAV9-mediated cardiomyocyte-specific knockdown of MYSM1 had a therapeutic effect on myocardial I/R injury. Using a comprehensive proteome-wide quantitative analysis, we identified STAT1 as a crucial substrate of MYSM1 in cardiomyocytes. Mechanistically, MYSM1 mediated the K63-linked deubiquitination and stabilization of STAT1 at position K379 via its MPN metalloprotease domain. Subsequently, MYSM1 initiated the expression of necroptosis-related genes by promoting the transcriptional activity of STAT1.

The JAMMs family represents the sole zinc metalloprotease family, while the remaining DUB families consist of cysteine peptidases 10. The distinctive catalytic mechanism of the JAMMs family makes them attractive candidates for drug development 10. Through comparing RNA-seq and functional screening of JAMMs, we identified MYSM1 as a potential regulator of myocardial I/R injury. Given that global deletion of MYSM1 in mice causes embryonic lethality 18, 19, we selected *Mysm1*^-/+^ mice to investigate the function of MYSM1 in myocardial I/R injury.* Mysm1*^-/+^ mice did not exhibit phenotypic abnormalities such as growth retardation, tail morphology, and abnormal blood parameters. In addition, mice suffering from myocardial I/R injury were protected by MYSM1 knockdown* in vivo*. Loss- and gain-of-function analysis further confirmed that MYSM1 promotes H/R-induced cardiomyocyte injury *in vitro*. Our study revealed that MYSM1 is a detrimental DUB in myocardial I/R injury and can be targeted pharmacologically for this condition. However, to date, there are no commercially available specific inhibitors targeting MYSM1 that warrant further development. On the other hand, MYSM1 plays a beneficial role in hematopoietic stem cell function and immune response 11-13. Consequently, the specificity of cardiac targeting must be taken into account when devising targeted inhibition strategies for MYSM1. Here, to specifically inhibit MYSM1 expression in cardiomyocytes, we utilized an AAV-9 vector containing a *cTNT* promoter driving shMYSM1 and demonstrated that cardiomyocyte-specific knockdown of MYSM1 protects against myocardial I/R injury. In summary, we propose that cardiomyocyte-targeted gene therapy utilizing AAVs encoding cardiac-specific promoters (such as *cTNT* or *Myh6*) could represent a promising therapeutic strategy for MYSM1-mediated myocardial I/R injury.

The role of DUBs is closely related to the function of their substrate proteins. The functions and substrates of MYSM1 vary across different pathophysiological processes 11. Through a comprehensive proteome-wide quantitative analysis, including interactomics and ubiquitinomics, we identified STAT1 as a direct substrate of MYSM1. We systematically elucidated the entire process of STAT1 deubiquitination modification by MYSM1. MYSM1 comprises three main domains: SANT, SWIRM, and MPN domain 11. The SANT domain of MYSM1 can bind to DNA and is required for MYSM1 to bind to histones 11, 13. The SWIRM domain of MYSM1 is essential for the interaction between MYSM1 and substrate proteins 11, 29. Consistently, our data showed that MYSM1 binds to STAT1 mainly through the SWIRM domain. The MPN metalloprotease domain is the crucial catalytic domain of MYSM1 11. In this study, we demonstrated that MYSM1 deubiquitinates and stabilizes STAT1 through the MPN metalloprotease domain of MYSM1. According to our ubiquitinomics, the K379 residue of STAT1, which is located in the DBD domain, is the most significantly reduced deubiquitination residue of STAT1 after MYSM1 overexpression. It has been reported that ubiquitination at K379 residue may inhibit the binding of STAT1 to gene promoter regions 30. Here, we found that MYSM1 increased transcriptional activity of STAT1, suggesting that MYSM1-mediated K379-deubiquitination of STAT1 may promote the promoter region binding of STAT1 to up-regulate gene transcription.

The irreversible cardiomyocyte death is a vital consequence of myocardial I/R injury 4. Given the non-regenerative capacity of cardiomyocytes 5, preventing PCDs is essential to alleviate myocardial I/R injury. Studies have demonstrated that STAT1 plays a significant role in PCDs 26, 27. STAT1-dependent regulation of PCD largely relies on its genomic occupancy on target genes 28. It has been reported that STAT1 promotes myocardial I/R injury and cardiomyocyte PCD 20, 21, while the transcriptional regulation mechanism behind remains unknown. Our findings revealed for the first time that nuclear STAT1 initiates the transcription of the necroptosis-related genes *Mlkl* and *Ripk3* by directly binding to their promoter regions. MYSM1 promotes the *Mlkl* and *Ripk3* expression by deubiquitinating and stabilizing STAT1. STAT1 plays different functions in various pathological states of the heart, such as a protective role in cardiac hypertrophy 31 but a pathogenic role in ischemic heart disease 26, 27, which makes it difficult to be a feasible target for heart diseases. Additionally, small molecule drugs targeting STAT1 often lack selectivity among STAT family members. In this study, we demonstrated a specific MYSM1-STAT1 axis in myocardial I/R injury, which might avoid the side effects of targeting STAT1 alone, indicating that targeting MYSM1 could be a more specific therapeutic strategy for myocardial I/R injury.

The functions and substrates of MYSM1 are different in various diseases. Apart from the MYSM1-STAT1 interaction, MYSM1 has been reported to deubiquitinate other substrate proteins 11, such as E2A 32, GATA2 33, and histone H2A 12. Although these substrates are less relevant to myocardial I/R injury and were not present in our comprehensive proteome-wide quantitative analysis, we cannot completely exclude the possibility that MYSM1 regulates myocardial I/R injury through these substrate proteins. In addition to STAT1, we also screen out some potential substrates from our comprehensive proteome-wide quantitative analysis, including *Actn4*, *Aldoa*, *Isg15*, *S100a4*, *Cct7* and *Lgals8*, which is worthy of further exploration.

In conclusion, we showed for the first time that MYSM1 deubiquitinates and stabilizes STAT1 to increase the transcription of necroptosis-related genes in cardiomyocyte under I/R injury. We also demonstrated the pathogenic role of specific MYSM1-STAT1 axis in myocardial I/R injury, suggesting that small molecule compounds targeting MYSM1 may be an attractive therapeutic strategy for this condition.

## Supplementary Material

Supplementary materials and methods, figures and tables.
