# Supplementary material for: Deubiquitinase MYSM1 drives myocardial ischemia/reperfusion injury by stabilizing STAT1 in cardiomyocytes
Source: Theranostics. 2025 Jan 2;15(5):1606–21. doi: 10.7150/thno.100097 (PMC11780537; doi:10.7150/thno.100097)
Supplement: Supplementary file 1 — Supplementary materials and methods, figures and tables. [file thnov15p1606s1.pdf]

Supplemental Material

Deubiquitinase MYSM1 drives myocardial ischemia/reperfusion injury by stabilizing STAT1 in cardiomyocytes

Supplementary Information:

1. Extend Materials and Methods
2. Supplementary Figure S1-6
3. Supplementary Table S1-8

Materials and Methods

Animal experiments

The whole-body *Mysm1*-knockdown mice (*Mysm1*$^{-/+}$, KD), *Mysm1*-knockout mice (*Mysm1*$^{-/-}$, KO, Strain NO. T058305) with a C57BL/6J background, and littermate wild-type (WT) mice were obtained from Gempharmatech. *Mysm1*$^{-/-}$mice exhibit partial embryonic lethality [1], growth retardation, as well as abnormalities in the hind limb and tail morphology [2], while *Mysm1*$^{-/+}$ mice show no phenotypic abnormalities. The adeno-associated virus serotype 9 (AAV-9), carrying a *cTNT* promoter driving short hairpin RNA (shRNA) targeting *Mysm1* gene sequence (CAGTGATGAGAACAGAGCTATCATT) was constructed by Shanghai Genechem Co. The *cTNT* AAV9-shMYSM1 (total dose 2×10^{11} vg/mouse) or negative control RNA (shNC) was administered to mice via tail vein injection. For the shRNA control, a scrambled sequence (TTCTCCGAACGTGTCACGT) was utilized for the generation of AAV9-shNC. After 4 weeks, the mice were subjected to myocardial I/R injury model.

For the myocardial I/R injury model, the left anterior descending (LAD) coronary artery was ligated and the animals were anaesthetized using 2% isoflurane. After opening the thorax at the fourth intercostal space of the left midclavicular line, the LAD artery was ligated for 30 min using 7-0 silk sutures. The presence of myocardial blanching in the perfusion bed confirmed complete occlusion of the vessel. The ligature was then untied, and reperfusion was initiated for either 4 hours (acute I/R injury) or 2 weeks (chronic I/R injury). After 2 weeks of reperfusion, cardiac function was assessed using transthoracic noninvasive echocardiography with a Vevo 3100 system equipped with an MS-550D ultrasound transducer (Fujifilm Visual Sonics). Isoflurane was used as an anesthetic during the echocardiography. All measurements derived from echocardiography were obtained by averaging the readings from three consecutive cardiac cycles. Subsequently, the mice were humanely euthanized under sodium pentobarbital anesthesia. Finally, blood and heart tissues were collected and stored for further analysis. Collected blood samples were transferred into EDTA-coated tubes and subsequently analyzed using an automated hematology analyzer (88 μL per sample, XN-1000, Sysmex). All animal care and experimental procedures strictly adhered to the Guide for the Care and Use of Laboratory Animals (US National Institutes of Health) and were approved by the ethics committee of the Second Affiliated Hospital of Jiaxing University (approval document no. JUMC2023-102).

Evans blue (EB)/ triphenyl tetrazolium chloride (TTC) staining

Hearts were cut into 1-mm thick transverse slices and stained for 15 min with EB and 2% TTC. The infarct (INF) size was determined by TTC staining, and the area-at-risk (AAR) area was defined by Evans blue dye. The images of stained sections were captured, and then the INF and AAR area were measured by planimetry with ImageJ software.

Detection of serum myocardial enzymes

Serum was collected after centrifugation to detect myocardium enzymes, including lactic dehydrogenase (LDH), creatine kinase-MB (CK-MB), and cardiac troponin T (cTnT). Following the manufacturer's instructions, serum LDH (A020-2-2) levels were determined using a

commercially available kit from Nanjing Jiancheng Bioengineering Institute. Serum CK-MB (E-EL-M0355c) and cTnT (E-EL-M1801c) levels were detected using their respective ELISA kits from Elabscience Biotechnology Company Limited. The Multiskan GO Microplate Reader (Thermo Fisher Scientific) was used to analyze the data spectrophotometrically.

Histological analysis and immunofluorescence staining

Cardiac tissues were fixed in 10% neutral-buffered formalin for 24 h at room temperature and embedded in paraffin. The sections (5 μm) were stained with hematoxylin and eosin (H&E, G1080, Solarbio) for histological analysis, Sirius Red (G1472, Solarbio) to evaluate the fibrosis content, and TUNEL (C1098, Beyotime) to assess apoptotic DNA fragmentation in heart tissues. All staining results were observed under a light microscope (Zeiss), and fibrotic areas were semi-quantitatively determined using ImageJ.

Frozen sections were used to perform immunofluorescence analysis for α-actinin-2, vimentin, F4/80 and MYSM1. Briefly, sections were incubated overnight at 4°C with primary antibodies for α-actinin-2 (7H1L69, Invitrogen, 1:200), vimentin (EM0401, Huabio, 1:200), F4/80 (sc-377009, Santa, 1:200), and MYSM1 (abs136708, Absin, 1:200). Homologous double labeling was achieved by a three-color multiple fluorescence immunohistochemical staining kit (abs50088, Absin). Alex 594-conjugated goat anti-rabbit (8889, CST, 1:200), iFluor™ 488 conjugated goat anti-rabbit (HA1121, HUABIO, 1:200) and Alex 488-conjugated goat anti-mouse (4408, CST, 1:200) antibodies were used as secondary antibodies. Nuclei were stained with DAPI (C0065, Solarbio). The stained sections were observed using a fluorescence microscope (Leica).

Cell culture, transfection and viability

As previously described, neonatal rat primary cardiomyocytes (NRPCs) were isolated from the ventricles of neonatal Sprague-Dawley rats [3]. The HL-1 and NIH/3T3 cells were obtained from the Shanghai Institute of Biochemistry and Cell Biology. HL-1 cells were cultured at 37°C under 5% CO_2 in Claycomb medium (51800C, Sigma) supplemented with 10% fetal bovine serum (FBS), 1% penicillin/streptomycin, 1% L-glutamine and Norepinephrine. NIH/3T3 cells were cultured at 37°C under 5% CO_2 in Dulbecco's modified eagle medium (DMEM, Solarbio) supplemented with 10% FBS and 1% penicillin/streptomycin. For the hypoxia/reoxygenation (H/R) model, oxygen-serum deprivation injury was induced by placing cells in a controlled hypoxia incubator (5% CO_2, 95% N_2) without serum medium for 4 h. Subsequently, the medium was replaced with normal culture media, and the cells were reoxygenated under normoxic conditions (21% O_2, 74% N_2, and 5% CO_2) for 6 h [4].

Overexpression of MYSM1, STAT1, and other proteins in HL-1 or NIH/3T3 cells was achieved using plasmids (Flag-MYSM1-WT/△MPN/△SWIRM/△SANT, Flag-BRCC3, Flag-COPS5, Flag-PSMD14, Flag-MPND, Flag-STAMBP, Flag-STAMBPL1, Flag-COPS6, Flag-PSMD7, Flag-EIF3H, Flag-EIF3F, Flag-CEBPB, Myc-STAT1-WT/K379R, and HA-Ub/K48/K63) provided by Genechem. Lipofectamine 3000 reagent (L3000-150, Thermo Fisher Scientific) was used to transfect the overexpression plasmids (1 μg plasmid/1 million cells) into the cells. MYSM1 small interfering RNA was constructed (siMYSM1: GCCAGACAAUACUUCAGAATT, Gene

Pharma Co. Ltd) to silence MYSM1 expression. The transfection agent Lipofectamine 2000 (11668-019, Thermo Fisher Scientific) was used to transfect siMYSM1 into NRPCs. The knockdown of MYSM1 and STAT1 in HL-1 cells was accomplished using lentiviral-based specific shRNA, constructed and purified by Hanbio Tech. The sequences of the shRNA were shown in **Table S1**. HL-1 cells were infected with the indicated lentiviruses, followed by selection with puromycin (10 μg/mL) for 2 weeks. Single stable cell lines were then generated.

Cell viability

Cell viability was assessed using the CCK8 assay (CA1210, Solarbio) according to the manufacturer's instructions. And cytotoxicity was assessed using the LDH release assay kit (C0016, Beyotime) according to the product manual.

Isolation of major cardiac cell types from mouse hearts

To isolate cardiomyocytes, EDTA buffer was infused into the right ventricle to clear the blood. The heart was sequentially treated with EDTA buffer, perfusion buffer, and collagenase buffer via injection into the left ventricle. The heart was carefully dissected into 1 mm fragments. Enzymatic activity was halted by adding a stop buffer. The resulting cell suspension was filtered through a 100 μm mesh and subjected to four rounds of gravity sedimentation using three intermediate buffers to progressively re-establish physiological calcium concentrations. One hour later, the medium was refreshed with pre-warmed culture medium. To isolate cardiac fibroblasts, heart tissues from 2-3-day-old C57BL/6 mice were cut into approximately 1 mm^3 pieces. The tissues were then dissociated using 0.07% collagenase II and 0.04% trypsin. The resulting cell suspension was filtered through a 70 μm cell strainer. The collected cells were resuspended and incubated in dishes for 1 hour to allow primarily cardiac fibroblasts to adhere to the plastic surface. To isolate cardiac endothelial cells, hearts were then dissected into pieces and enzymatically digested using an enzyme kit (130-110-201, Miltenyi) to obtain a single-cell suspension. The resulting suspension was filtered through 70 μm strainers to eliminate cell aggregates and incubated with CD31 beads (130-097-418, Miltenyi). After washing, the endothelial cells were purified using an LD Column (130-042-901, Miltenyi). To isolate cardiac macrophages, hearts were rinsed in warmed Hanks' Balanced Salt Solution, and then minced and incubated in a warm digestion buffer containing Liberase TH and DNase I. The mixture was triturated with a pipette and filtered through a 40 μm sieve into ice-cold stopping buffer composed of 10% FBS. This procedure was repeated 5 times. The cells were centrifuged, the supernatant was discarded, and red blood cells were lysed with ACK lysing buffer (A10492-01, Gibco). Next, the cells were resuspended in HBSS and incubated with 50 μl of anti-CD45 magnetic beads for 30 minutes (130-052-301, Miltenyi). The labeled cells were obtained by a MACS magnetic column. Specific markers are used to verify the purity of each cell type, such as α-actinin for cardiomyocytes, vimentin for fibroblasts, CD31 for endothelial cells and F4/80 for macrophages.

Propidium iodide (PI) determination

The measurement of PI was performed using Hoechst and PI double fluorescence staining (C1056,

Beyotime). After treatment, the cells were stained with Hoechst and PI for 30 minutes. The cells were then examined under a fluorescence microscope (Leica).

Interactomics and ubiquitinomics analysis

Interactomics analysis: to investigate MYSM1-binding proteins in cells, HL-1 cells were transfected with Flag-MYSM1 or empty vehicle (EV) plasmids. Cells were harvested using lysis buffer and incubated with anti-Flag-beads (B26102, Bimake) in 4 °C overnight. After five washes, the precipitated protein mixtures were added to SDT lysate (4% SDS, 100 mM DTT, 100 mM TrisHCl) for enzymatic hydrolysis and subsequently desalted using C18 Stage Tip. The samples were then subjected to liquid chromatography-tandem mass spectrometry (LC-MS/MS) analysis (Thermo Scientific). The MS data were analyzed using MaxQuant software version 2.0.1.0. The calculation of the score value is embedded within MaxQuant. The data were searched against the UniProt reference proteome Mus musculus database (55,286 total entries). Trypsin was selected as the digestion enzyme, allowing for a maximum of two missed cleavages. The mass tolerance was set to 4.5 ppm for precursor ions and 20 ppm for fragment ions. Carbamidomethylation of cysteines was specified as a fixed modification, while acetylation of protein N-termini and oxidation of methionine were set as variable modifications. The database search results were filtered and exported with a false discovery rate (FDR) of <1% at both the peptide-spectrum match and protein levels.

Ubiquitinomics analysis: HL-1 cells were transfected with Flag-MYSM1 or EV plasmids. Following digestion, the peptides obtained for ubiquitin peptide enrichment were dissolved in NETN buffer and incubated with anti-diglycine remnant (K-ε-GG, 5562, CST) pan antibody beads with gentle shaking (4 °C, overnight). The precipitated peptide mixtures were then subjected to LC-MS/MS analysis. The MS data were analyzed using Proteome Discoverer 2.4. The data were searched against the UniProt Mus musculus database (88,473 total entries). Tryptic cleavage specificity was applied, with variable modifications including oxidation (M), acetylation (protein N-terminus), deamidation (NQ), and Glygly (K), and fixed carbamidomethyl cysteine modification. Peptides were required to have a minimum length of 6 amino acids, and each protein had to be identified by at least one unique peptide. For peptide and protein identification, the FDR was set to 1%. Site quantitation analysis was filtered to include only those phosphorylation sites with a localization probability of ≥0.75, as determined by the algorithm. Label-free quantification was performed using intensity-based methods, and the quantitative site ratios were weighted and normalized by the median ratio. The proteomics raw data have been uploaded to the ProteomeXchange dataset via the PRIDE repository (Accession: PXD058830).

Western blotting and co-immunoprecipitation (Co-IP) assay

Using lysis buffer (AR0103/AR0101, Boster), proteins were extracted from cultured cells or tissues. Protein concentration was determined by the BCA protein assay. Protein lysates were separated using SDS-PAGE and transferred to PVDF membranes. Membranes were blocked with 3% bovine serum albumin (BSA) for 2 h at room temperature and subsequently incubated overnight at 4°C with the following primary antibodies: MYSM1 (abs136708, **Absin**, 1:1000), STAT1 (14994, CST,

1:1000), p-STAT1-Tyr701 (7649, CST, 1:1000), Flag (20543-1-AP, Proteintech, 1:1000), HA (51064-2-AP, Proteintech, 1:1000), JAK1 (abs146102, Absin, 1:1000), p-JAK1 (abs173033, Absin, 1:1000), and SHP2 (abs113519, Absin, 1:1000), LaminB (abs159524, Absin, 1:1000), RIPK3 (17563-1-AP, Proteintech, 1:1000), MLKL (66675-1-Ig, Proteintech, 1:1000), p-MLKL (37333**,** CST, 1:1000), TUBULIN (2128, CST, 1:1000), and GAPDH (5174, CST, 1:1000). Then, the membranes were incubated for 2 h at room temperature with the following secondary antibodies: goat anti-rabbit secondary antibody (7074, CST, 1:5000) or goat anti-mouse secondary antibody (7076, CST, 1:5000). The ECL detection kit (WBKLS0500, Merck Millipore) was used to visualize protein expression levels on the Bio-Rad ChemiDoc XRS$^+$ Imaging Systems (Bio-Rad).

Co-IP involved the isolation of proteins from cultured cells or tissues using lysis buffer, with part of the lysis solution being retained as the input sample. Other lysates were incubated with respective primary antibody or the anti-Flag-beads (B26102, Bimake) for MYSM1 and anti-Myc-beads (B26102, Bimake) for STAT1 (overnight, 4°C). Protein A+G agarose beads (P2055, Beyotime) were then added to the protein lysis solution (4 h, 4°C). After three washes with IP lysis buffer, the protein-bead mixtures were employed for subsequent western blot experiments.

RT-qPCR

Trizol reagent (15596018CN, Invitrogen) was used to isolate the total RNA from HL-1 cells and heart tissues. After purifying the RNA, the Prime Script RT Master Mix reagent (RR036A, Takara) was used to create the cDNA. Then, the cDNA was used for quantitative RT-PCR by using SYBR Green^{TM} Premix Ex Taq^{TM} II (RR820A, Takara). The amount of each gene was determined and normalized to the amount of *β-actin*. Primer sequences were detailed in **Table S2**.

CUT&Tag assays

HL-1 cells were transfected with Flag-MYSM1 expressing plasmids (oeMYSM1) or EV plasmids before H/R treatment. The CUT&Tag assay was conducted following the manufacturer's protocol with the NovoNGS CUT&Tag 4.0 HighSensitivity Kit (Novoprotein). In brief, NovoNGS ConA Beads were washed with ConA Binding Buffer. 1x10^5 cells were prepared and immobilized on concanavalin A beads. Cells with beads were incubated overnight at 4 °C with STAT1 antibody (1:50). Subsequently, the goat anti-rabbit IgG secondary antibody (1:100, N269-01A, Novoprotein) was added into the sample at room temperature for 1.5 h. After removing the unbound secondary antibody, cells were incubated with NovoNGS ChiTag (pAG-Transposome) for 1 h at room temperature. The samples were washed with ChiTag Buffer and then subjected to tagmentation using Tagmentation Buffer. Reactions were stopped by the addition of 5 μL Stop Buffer and 1 μL Proteinase K to each sample and incubated at 55 °C for 10 min. DNA was isolated by NovoNGS DNA Clean Beads and dissolved in TE-RA-Buffer. For high-throughput sequencing and qPCR assays, DNA was amplified with N5 and N7 primers and purified with NovoNGS DNA Clean Beads (Primer sequences were listed in **Table S3**).

Luciferase reporter gene assay

Cells were co-transfected with STAT1, *Mlkl* or *Ripk3* promoter labeled with luciferase reporters

(Hanbio, Shanghai, China), and renilla luciferase with Lipofectamine 3000 reagent (L3000-150, Thermo Fisher Scientific). After transfection for 48 h, the firefly and renilla luciferase activities were measured by Dual-Luciferase Reporter Assay kit (HB-DLR-100, Hanbio, Shanghai, China) using a Varioskan LUX Multimode Microplate Reader (Thermo), and the ratio of firefly/renilla luciferase activity was calculated.

Statistical analysis

Data from both *in vitro* and *in vivo* experiments were expressed as mean ± standard error of the mean (SEM). An unpaired Student's t-test was performed to compare two samples, while ANOVA followed by Bonferroni's Tukey's post hoc test was used to compare multiple samples (GraphPad Pro Prism 8.0). Statistical significance was defined as $P < 0.05$.

Reference

1. Fiore A, Liang Y, Lin YH, Tung J, Wang H, Langlais D, et al. Deubiquitinase MYSM1 in the Hematopoietic System and beyond: A Current Review. Int J Mol Sci. 2020; 21.
2. Nijnik A, Clare S, Hale C, Raisen C, McIntyre RE, Yusa K, et al. The critical role of histone H2A-deubiquitinase Mysm1 in hematopoiesis and lymphocyte differentiation. Blood. 2012; 119: 1370-9.
3. Ye B, Shi X, Xu J, Dai S, Xu J, Fan X, et al. Gasdermin D mediates doxorubicin-induced cardiomyocyte pyroptosis and cardiotoxicity via directly binding to doxorubicin and changes in mitochondrial damage. Transl Res. 2022; 248: 36-50.
4. Zhong L, Han J, Fan X, Huang Z, Su L, Cai X, et al. Novel GSDMD inhibitor GI-Y1 protects heart against pyroptosis and ischemia/reperfusion injury by blocking pyroptotic pore formation. Basic Res Cardiol. 2023; 118: 40.

Supplementary Figures:

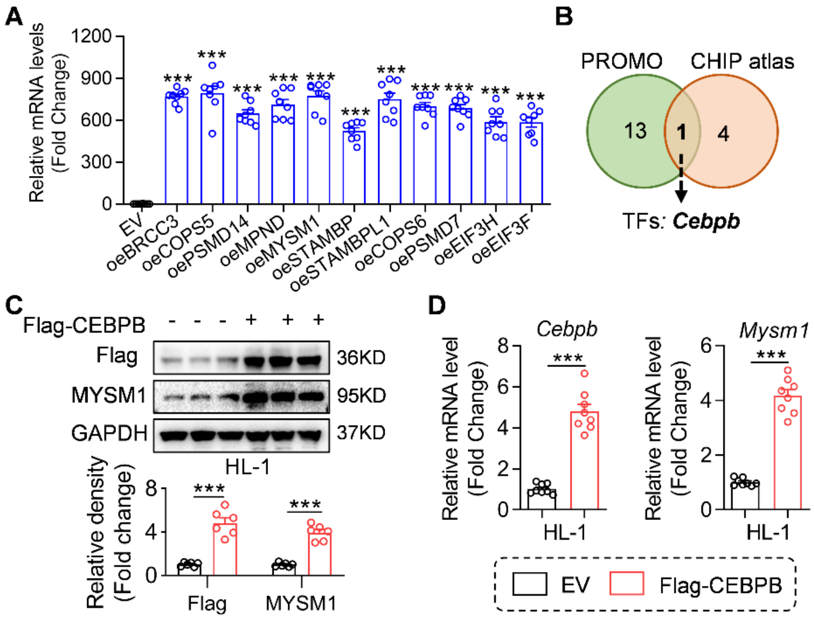

Figure S1. A. The overexpression efficiency of JAMMs family plasmids in HL-1 cells was verified by RT-qPCR (n = 8; ***, $P < 0.001$ vs. EV). **B.** Comprehensive combined PROMO database and CHIP atlas for identification of CEBPB as a transcription factor of MYSM1. **C-D.** We transfected with Flag-CEBPB or empty vehicle (EV) plasmid in HL-1 cells. (**C**) Representative western blotting analysis of Flag-CEBPB and MYSM1 in HL-1 cells, with protein level standardized by GAPDH (n = 6)**.** (**D**) RT-qPCR analysis of mRNA level of *Cebpb* and *Mysm1* (n = 8). (** $P < 0.01$, *** $P < 0.001$).

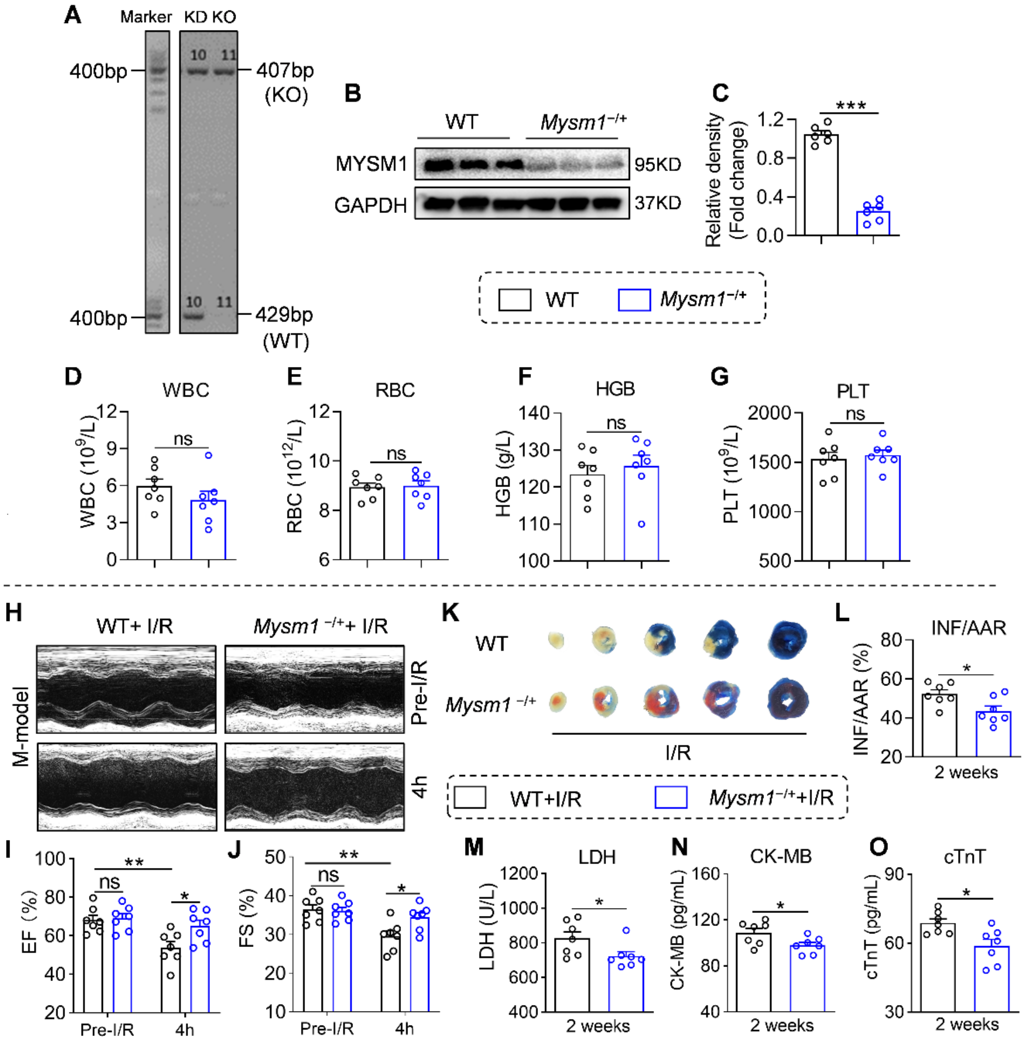

Figure S2. A. PCR using WT and *Mysm1*$^{-/-}$ (KO) primers to identify the genotype of mice. The PCR product length of WT and KO primers were 429 bp and 407 bp, respectively. **B-C.** Representative western blotting analysis of MYSM1 in heart tissues, with protein level standardized by GAPDH (n = 6). **D-G.** Bar graphs showing the level of white blood cells (WBC, **D**), red blood cells (RBC, **E**), hemoglobin (HGB, **F**), and platelets (PLT, **G**) in WT and the *Mysm1*$^{-/+}$ mice (n = 7). **H-J**. WT and *Mysm1*$^{-/+}$ mice subjected to acute I/R injury (30 min of ischemia and 4 h of reperfusion). (**H**) Mice M-mode echocardiography images from each group. (**I-J**) Relative levels of ejection fraction (EF%, **I**) and fractional shortening (FS%, **J**) in mice (n = 7). **K-O**. WT and *Mysm1*$^{-/+}$ mice subjected to chronic I/R injury (30 min of ischemia and 2 weeks of reperfusion). (**K-L**) The Myocardial infarction size determined by EB/ TTC double-staining and quantitative data for INF (**L**). (**M-O**) The levels of serum LDH (**M**), CK-MB (**N**), and cTnT (**O**) following chronic myocardial I/R injury (n = 7). (ns = no significance, * $P < 0.05$, ** $P < 0.01$, *** $P < 0.001$).

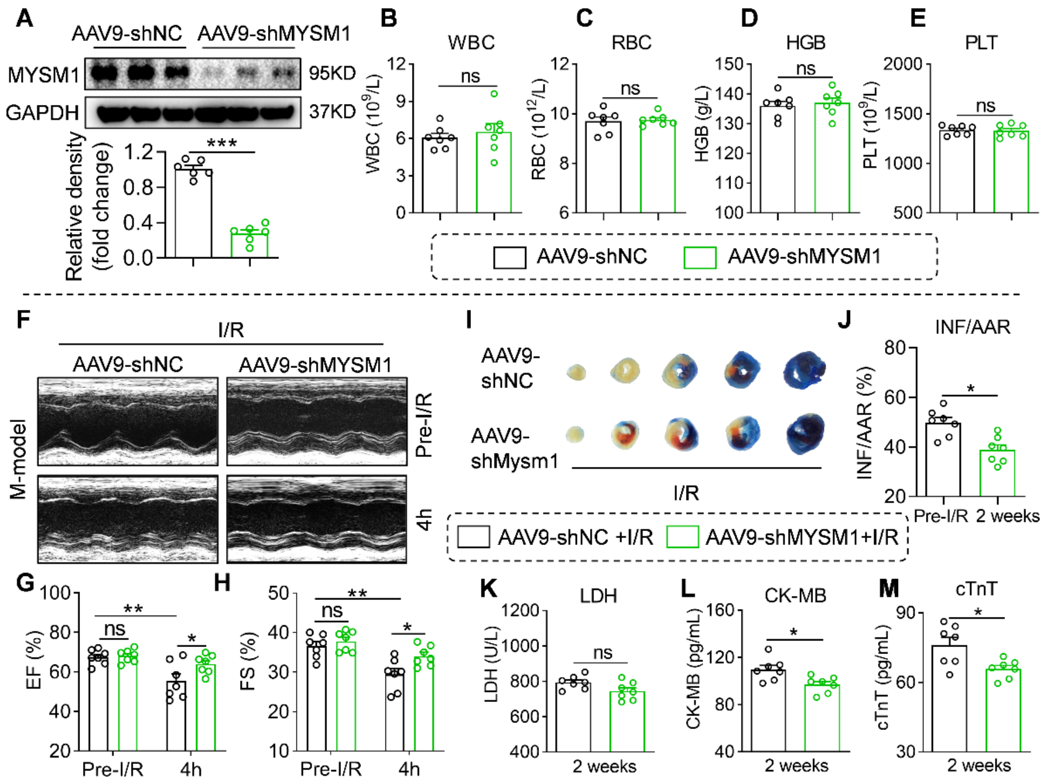

Figure S3. A. Representative western blotting analysis of MYSM1 in heart tissues, with protein level standardized by GAPDH (n = 6). **B-E.** Bar graphs showing the levels of WBC (**B**), RBC (**C**), HGB (**D**), and PLT (**E**) in AAV9-shNC and AAV9-shMYSM1 mice (n = 7). **F-H**. AAV9-shNC and AAV9-shMYSM1 mice subjected to acute I/R injury (30 min of ischemia and 4 h of reperfusion). (**F**) Mice M-mode echocardiography images from each group. (**G-H**) Relative levels of EF% (**G**) and FS% (**H**) in mice (n = 7). **I-M**. AAV9-shNC and AAV9-shMYSM1 mice subjected to chronic I/R injury (30 min of ischemia and 2 weeks of reperfusion). (**I-J**) The Myocardial infarction size determined by EB/ TTC double-staining and quantitative data for INF (**J**). (**K-M**) The levels of serum LDH (**K**), CK-MB (**L**), and cTnT (**M**) following chronic myocardial I/R injury (n = 7). (ns = no significance, * $P < 0.05$, ** $P < 0.01$, *** $P < 0.001$).

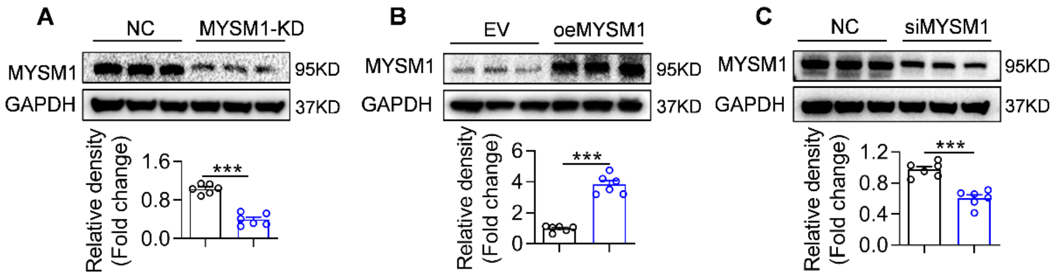

Figure S4. A. The inhibition efficiency of MYSM1-knockdown (KD) HL-1 cells verified by western blotting, with protein level standardized by GAPDH (n = 6). **B**. The overexpression efficiency of oeMYSM1 plasmid in HL-1 cells verified by western blotting, with protein level standardized by GAPDH (n = 6). **C.** The inhibition efficiency of siMYSM1 in HL-1 cells verified by western blotting, with protein level standardized by GAPDH (n = 6). (*** $P < 0.001$).

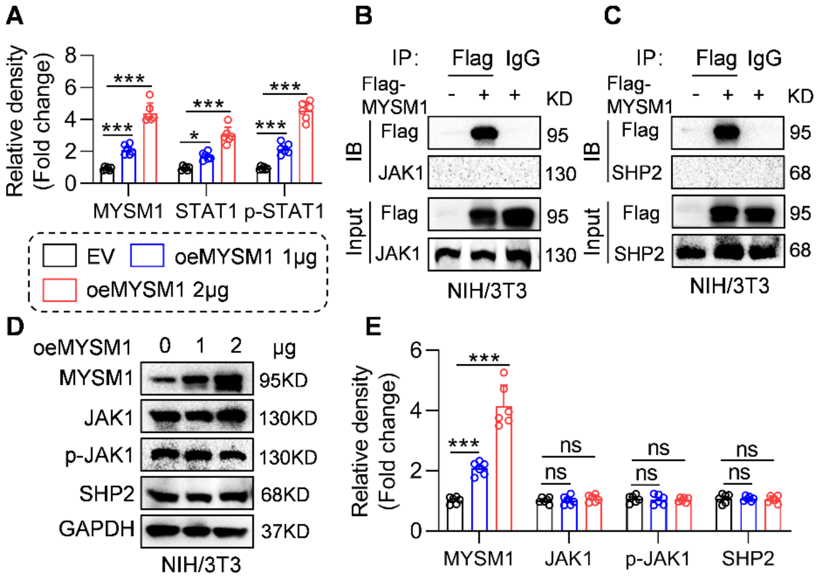

Figure S5. A. Representative western blotting for MYSM1, STAT1, p-STAT1 and GAPDH (loading control) in NIH/3T3 cells (**Fig.6A**) and the densitometric quantification (**A**, n = 6). **B-C**. CO-IP of Flag-MYSM1 in NIH/3T3 cells overexpressed with Flag-MYSM1. Exogenous Flag-MYSM1 was immunoprecipitated by anti-Flag antibody. (**B**) Western blot detected the protein levels of Flag-MYSM1 and JAK1. (**C**) Western blot detected the protein levels of Flag-MYSM1 and SHP2. **D-E.** NIH/3T3 cells were transfected with Flag-MYSM1 (1 or 2 μg) then analyzed by western blot for MYSM1, JAK1, p-JAK1, SHP2 and GAPDH (loading control) (n = 6). (ns = no significance, * $P < 0.05$, ** $P < 0.01$, *** $P < 0.001$).

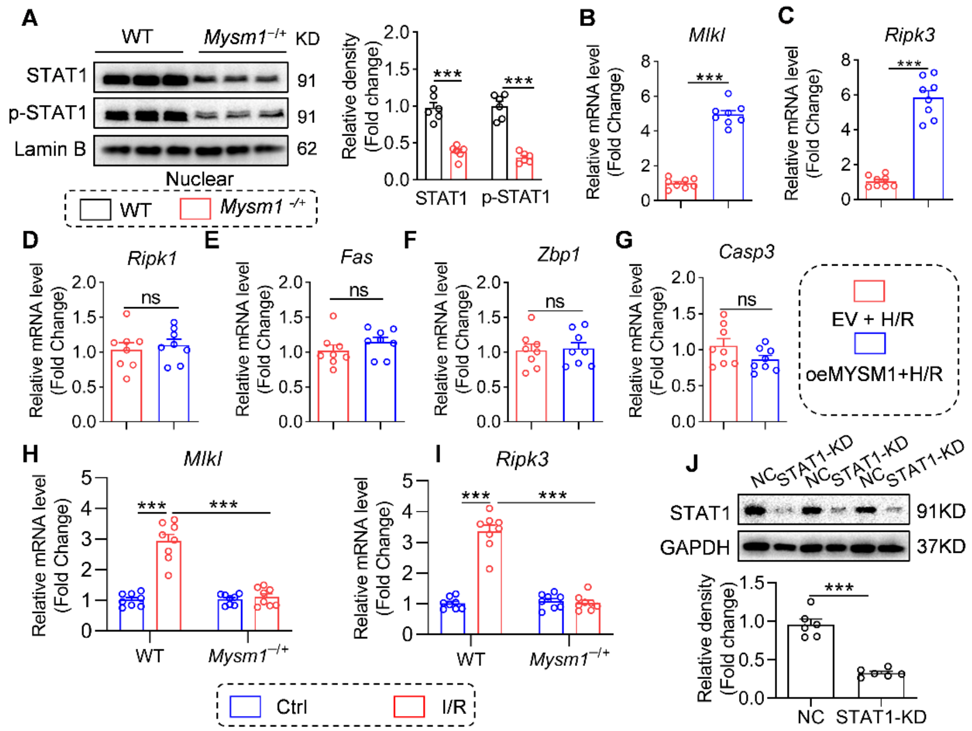

Figure S6. A. Representative western blotting for STAT1, p-STAT1 (Tyr701) and Lamin B (loading control) in heart tissues and densitometric quantification (n = 6). **B-G.** HL-1 cells were transfected with oeMYSM1or EV plasmid before H/R injury. RT-qPCR analysis of mRNA levels of *Mlkl* (**B**), *Ripk3* (**C**), *Ripk1* (**D**), *Fas* (**E**), *Zbp1* (**F**), and *Casp3* (**G**) (n = 8). **H-I.** The mRNA levels of *Mlkl* (**H**) and *Ripk3* (**I**) in myocardial I/R injury were detected by RT-qPCR (n = 8). **J.** The inhibition efficiency of STAT1-knockdown (KD) HL-1 cells verified by western blotting (n = 6). (ns = no significance, * $P < 0.05$, ** $P < 0.01$, *** $P < 0.001$).

Supplementary Tables:

Table S1. Sequences of shRNA

**Gene/ Species**	**Top strand**	**Bottom strand**
Mysm1/ Mouse	5'-GATCCGCAGTGATGAGAACAGAG CTATCATTCTCGAGAATGATAGCTCT GTTCTCATCACTGTTTTTTG-3'	5'-AATTCAAAAAACAGTGATGAGA ACAGAGCTATCATTCTCGAGAATGA TAGCTCTGTTCTCATCACTGCG-3'
Stat1/ Mouse	5'- GATCCGCTGTTACTTTCCCAGATA TTCTCGAGAATATCTGGGAAAGTAA CAGCTTTTTTG-3'	5'- AATTCAAAAAAGCTGTTACTTTC CCAGATATTCTCGAGAATATCTGGG AAAGTAACAGCG-3'

Table S2. Primers for RT-qPCR

**Gene name**	**Species**	**Sequence (5' to 3')**
Mysm1	Mouse	F: TGCACAGCCGGGAAATGAT<br>R: ATGGTGCTATCCAGAGTCCAA
Cebpb	Mouse	F: CGCCGCCTTATAAACCTCCC<br>R: AGTCGGGCTCGTAGTAGAAGT
β-Actin	Mouse	F: CTCTCCCTCACGCCATC<br>R: ACGCACGATTTCCCTCTC
Irf1	Mouse	F: GAAGGGAAGATAGCCGAAGAC<br>R: TCTGGTTCCTCTTTGCAGC
Mlkl	Mouse	F: GATTGCCCTGAGTTGTTGCG<br>R: CGTGGATTCTTCAACCGCAG
Ripk3	Mouse	F: CTCCGTGCCTTGACCTACTG<br>R: TCACCAGAGGAACCGCATAAC
Ripk1	Mouse	F: CTAGCACCACCCCAGTAGTC<br>R: TCGGAGTTCGGTGCTGAAG
Casp3	Mouse	F: TGGTGATGAAGGGGTCATTTATG<br>R: TTCGGCTTTCCAGTCAGACTC
Fas	Mouse	F: CAAGGAGGCCCATTTTGCTG<br>R: CCTGCAATTTCCGTTTGGCT
Zbp1	Mouse	F: AAGAGTCCCCTGCGATTATTTG<br>R: TCTGGATGGCGTTTGAATTGG

Table S3. Primers for CUT&Tag qPCR analysis

Gene name	Species	Sequence (5' to 3')
Irf1	Mouse	F: AAGGGTGAATCGTAGCAGTGG<br>R: AAAGTCAGCCTGTGTCCTGG
Mlkl	Mouse	F: TTGGCCTCGAACTCACAGAG<br>R: GTGCTGAGGCGGTTCCTTTA
Ripk3	Mouse	F: TGCACCACCAGTCCTGTTAC<br>R: GGTAAGGTATGAGGCACGCA

Table S4.

**Predicated transcription factors from PROMO database**			
Fos	**Cebpb**	Jund	Hes1
Nf1	Cebpa	Yy1	F(alpha)-f(epsilon)
Gsr	Jun	Nfkb	Hoxa5
Myod	Ap1		

Factors predicted within a dissimilarity margin less or equal than 1%.

Table S5

**Reported TFs in cardiomyocytes from CHIP atlas**		
Foxo1	**CEBPB**	P300
Nkx2.5	Gata4	

Table S6. Echocardiographic parameters in *Mysm1*$^{-/+}$mice after I/R injury

	Pre-I/R		Reperfusion 2 weeks	
Parameter	WT+I/R (n=7)	*Mysm1*$^{-/+}$+I/R (n=7)	WT+I/R (n=7)	*Mysm1*$^{-/+}$+I/R (n=7)
Heart Rate (bpm)	412.262 ± 63.028	405.283 ± 28.287	414.170 ± 34.986	418.763 ± 69.226
LVIDs (mm)	2.131 ± 0.157	2.260 ± 0.159	3.070 ± 0.163*	2.311 ± 0.229#
LVIDd (mm)	3.432 ± 0.258	3.587 ± 0.195	3.702 ± 0.446	3.212 ± 0.578#
LVAWs (mm)	1.240 ± 0.148	1.113 ± 0.110	1.134 ± 0.164	1.223 ± 0.144
LVAWd (mm)	0.735 ± 0.101	0.744 ± 0.107	0.749 ± 0.114	0.720 ± 0.108
IVSs (mm)	1.112 ± 0.132	1.106 ± 0.125	0.798 ± 0.125*	1.002 ± 0.129#
IVSd (mm)	0.703 ± 0.139	0.694 ± 0.122	0.731 ± 0.093	0.785 ± 0.127

LVID, left ventricular internal diameter; d, diastole; s, systole; LVAW, left ventricular anterior wall; IVS, interventricular septum (*, vs WT+I/R of Pre-I/R; #, vs WT+I/R of Reperfusion 2 weeks; # P<0.05, * P<0.05).

Table S7: Echocardiographic parameters in AAV9-shMYSM1 mice after I/R injury

	Pre-I/R		Reperfusion 2 weeks	
Parameter	AAV9-shNC+I/R (n=7)	AAV9-shMYSM1+I/R (n=7)	AAV9-shNC+I/R (n=7)	AAV9-shMYSM1+I/R (n=7)
Heart Rate (bpm)	401.281 ± 31.405	414.669 ± 41.647	402.297 ± 34.376	425.107 ± 32.866
LVIDs (mm)	2.087 ± 0.116	2.183 ± 0.214	2.989± 0.182*	2.391± 0.320#
LVIDd (mm)	3.381 ± 0.159	3.310 ± 0.318	3.731± 0.413	3.201± 0.200#
LVAWs (mm)	1.105 ± 0.169	1.102 ± 0.081	1.112 ± 0.144	1.202 ± 0.130
LVAWd (mm)	0.724 ± 0.091	0.725 ± 0.100	0.782 ± 0.119	0.762 ± 0.181
IVSs (mm)	1.092 ± 0.184	1.116 ± 0.181	0.804± 0.078*	1.029 ± 0.216#
IVSd (mm)	0.701 ± 0.112	0.711 ± 0.121	0.726 ± 0.094	0.727 ± 0.100

LVID, left ventricular internal diameter; d, diastole; s, systole; LVAW, left ventricular anterior wall; IVS, interventricular septum (*, vs AAV9-shNC +I/R of Pre-I/R; #, vs AAV9-shNC+I/R of Reperfusion 2 weeks; # P<0.05, * P<0.05).

Table S8: STAT1 peak annotation results from CUT&Tag-sequence

Gene Name	annotation	fold enrichment	-LOG10	chr	start	end	length
Mlkl	Promoter	9.64236	6.44644	NC_000074.7	112064560	112064915	356
Mlkl	Promoter	6.6548	4.83483	NC_000074.7	112063250	112063507	258
Mlkl	Promoter	5.25611	4.02903	NC_000074.7	112064091	112064298	208
Ripk3	Promoter	9.64236	6.44644	NC_000080.7	56027891	56028091	201
Ripk1	Promoter	14.4949	8.86385	NC_000079.7	34186097	34186736	640
Fas	Promoter	6.6548	4.83483	NC_000085.7	34267836	34268136	301
Zbp1	Promoter	5.25611	4.02903	NC_000068.8	173059893	173060185	293
Zbp1	Promoter	14.4949	8.86385	NC_000068.8	173060437	173061521	1085
Casp3	Promoter	14.4949	8.86385	NC_000074.7	47070005	47071098	1094
